# Exploratory Mendelian Randomization Provides Hypothesis-Generating Evidence for a Possible Nicotine-Related Mechanism in Sarcoidosis

**DOI:** 10.3390/biomedicines14102346

**Published:** 2026-10-15

**Authors:** Qian Yao, Mengmeng Zhao, Keting Min, Kun Peng, Ying Zhou

**Affiliations:** 1 Department of Respiratory Medicine, Shanghai Pulmonary Hospital, Tongji University School of Medicine, Shanghai 200433, China; 2 Department of Clinical Research Management, Shanghai Pulmonary Hospital, Tongji University, Shanghai 200433, China; 3 Clinical Research and Development Center of Shanghai Municipal Hospitals, Shanghai Hospital Development Center, Shanghai 200040, China

**Keywords:** sarcoidosis, nicotine, smoking, multivariable Mendelian randomization study

## Abstract

**Objective**: We explored whether the inverse association between smoking and sarcoidosis observed in observational studies may involve nicotine-related mechanisms. **Methods**: Genetic summary statistics were sourced from the largest available genome-wide association studies, covering smoking initiation, age of onset, daily cigarette consumption (CigDay), smoking cessation, lifetime smoking exposure, and nicotine metabolite ratio (NMR). Multivariable Mendelian randomization (MVMR) estimated the association of genetically predicted NMR with sarcoidosis risk after adjusting for CigDay, with univariable MR (UVMR) as supportive evidence. **Results**: Higher NMR was associated with higher sarcoidosis risk after adjusting for CigDay (OR = 1.08, 95% CI: 1.01–1.16, *p* = 0.018). UVMR showed that current smokers had lower risk than former smokers (OR = 0.44, 95% CI: 0.25–0.76, *p* = 0.003), whereas associations for current and former smoking versus never smoking, and for nicotine metabolites, were null. **Conclusions**: This exploratory MR study provides hypothesis-generating evidence that genetically predicted faster nicotine metabolism may be associated with higher sarcoidosis risk, compatible with a possible role of nicotine-related mechanisms but not establishing a causal effect or a clinical effect of nicotine. These results should not justify smoking, which remains strongly harmful. Future studies should investigate the immune regulatory pathways through which nicotine metabolism may influence sarcoidosis pathogenesis.

## 1. Introduction

Sarcoidosis is an immune-mediated granulomatous disorder that can affect any organ, with the lungs being the most commonly involved site [1]. The disease is characterized by macrophage release of pro-inflammatory factors and the formation of granulomas within a polarized T-helper 1 (Th1) immune milieu [2]. Despite its ambiguous cause, sarcoidosis is believed to stem from an overactive immune reaction to certain environmental factors in patients with genetic predispositions [3]. Smoking is linked to various autoimmune disorders, potentially acting as an environmental trigger [4].

Notably, among the numerous components of tobacco smoke, nicotine is of particular interest due to its potential protective role against sarcoidosis, possibly through the suppression of Th1-driven immune responses [5]. In line with this, several observational studies have reported an inverse association between smoking and sarcoidosis, although the underlying mechanisms remain unclear [6,7]. In one trial involving 13 patients with active pulmonary sarcoidosis (stages II or III), along with 6 asymptomatic and 6 disease-free controls, a 12-week course of nicotine-patch therapy (21 mg daily) showed no clinical benefit, although it did lead to an increase in regulatory T cells (Tregs) and a restoration of Toll-like receptors (TLR) 2 and 9, both implicated in sarcoidosis pathogenesis [8]. Another study with a similar design but extending over 24 weeks reported a significant improvement in forced vital capacity (FVC) compared to the control group [9]. Given the small scale of these studies, alongside confounding variables and potential reverse causality, the role of smoking in sarcoidosis remains unclear. Moreover, randomized controlled trials (RCTs) on nicotine’s long-term effects in sarcoidosis might unintentionally expose the control group to nicotine, raising addiction risks.

In this study, multivariable Mendelian randomization (MVMR) was used to estimate the association of genetically predicted nicotine metabolism with sarcoidosis risk after adjusting for cigarette consumption, with univariable MR (UVMR) included as a supportive, hypothesis-generating analysis to estimate the overall effect of smoking. These exploratory findings provide hypothesis-generating evidence for a possible nicotine-related mechanism in sarcoidosis.

## 2. Materials and Methods

### 2.1. Data Sources

This study utilized publicly available GWAS summary data. Data on smoking behaviors were sourced from the Sequencing Consortium of Alcohol and Nicotine use (GSCAN) [10], while a composite lifetime smoking index (LifeSmk) was derived from the UK Biobank [11]. Descriptions of the four smoking traits, smoking initiation (SmkIni), age of smoking initiation (AgeSmk), daily cigarette consumption (CigDay), and smoking cessation (SmkCes), are provided in the Supplementary Materials S1. Nicotine metabolite ratio (NMR), defined as the ratio of 3′-hydroxycotinine (3H) to cotinine (COT), reflects the rate of nicotine metabolism [12]. A high NMR indicates that under identical nicotine exposure, the human body retains less nicotine and eliminates it more rapidly. Summary statistics for NMR were obtained from the largest GWAS of current smokers [13]. Data for sarcoidosis were obtained from the FinnGen consortium [14]. Sarcoidosis cases were individuals with at least one recorded code of sarcoidosis in the Finnish Hospital Discharge Register or the Cause of Death Register (ICD-10 D86; ICD-9 135; ICD-8 135) or with a KELA special reimbursement code 132, without sex restriction or a minimum number of events. The endpoint thus represents clinically diagnosed sarcoidosis at any anatomical site as coded in Finnish national registers. The exposure GWAS datasets and FinnGen are independent consortia, so that any potential overlap, if present, would be small.

### 2.2. Mendelian Randomization and Statistical Analysis

For UVMR, the inverse variance weighted (IVW) method was used as the primary analysis to estimate odds ratio (OR) and 95% confidence interval (95% CI). A Bonferroni-corrected significance threshold of *p* < 0.01 (0.05/5 exposures) was applied for multiple testing. To assess the stability of the UVMR results, three sensitivity analysis approaches, MR-Egger, weighted median, and MR-PRESSO, were employed. Heterogeneity was assessed by Cochran’s Q test and quantified by the I^2^ statistic, where values of 25%, 50%, and 75% indicated low, moderate, and high heterogeneity, respectively [15]. A leave-one-out analysis was conducted by sequentially excluding individual SNPs to examine their influence on the overall MR estimates. The MR Steiger directionality (MRSD) test confirmed that the causal direction was from exposure to outcome. For each SNP, the F-statistic was calculated as previously described [16], and the power of the causal estimate was assessed using an online tool (https://shiny.cnsgenomics.com/mRnd/ (accessed on 5 October 2026)).

The primary MVMR analysis used the MVMR-IVW method, with the MVMR-weighted median, MVMR-Egger, MVMR-LASSO method applied as a supplementary analysis to assess the association of genetically predicted NMR with sarcoidosis [17]. For the primary MVMR model, we estimated causal effects for two pre-specified exposures (NMR and CigDay) simultaneously within a single joint regression framework. While Bonferroni correction remains valid for correlated tests, it tends to produce overly conservative inference under strong statistical dependence. We therefore applied a two-sided significance threshold of α = 0.05 for the causal estimates. Instrument robustness and validity were evaluated using the conditional F-statistic and Cochran Q test [18,19]. In the MVMR analysis, the conditional F-statistic measures the predictive strength of each exposure after adjusting for the effects of other variables [19]. According to established thresholds, the F-statistic should be >10 for strong instruments, and the Q-statistic should be less than the number of genetic variants for acceptable heterogeneity [20,21]. The non-zero MVMR-Egger intercept indicates the presence of directional pleiotropy. Leave-one-out analysis was performed to assess whether any single variant disproportionately influenced the causal estimates and an SNP-level scatter plot was generated to enable visual inspection of potential horizontal pleiotropy and outlier variants. A post-hoc power estimate for the primary NMR-sarcoidosis analysis was computed conditional on the observed effect size and standard error, noting that such post-hoc power calculations are largely driven by the observed data and provide limited additional inferential value. The R script for this power calculation is provided in Supplementary Materials S2.

The sensitivity analyses explored the association of genetically predicted current tobacco smoking (current vs. never smokers, CurSmk), past tobacco smoking (former vs. never smokers, PastSmk), and nicotine metabolite (COT, COT+3H) with sarcoidosis in UVMR. The trait “height” was analyzed as a negative control exposure (NCE) to assess population stratification bias from smoking status misalignment in the MVMR analysis of NMR and CigDay given its established biological irrelevance to sarcoidosis [22], null genetic correlation with smoking status in European-ancestry populations [23], and consistent ancestral matching with the primary exposures and outcome.

All statistical analyses were performed using the TwoSampleMR, MVMR, MendelianRandomization, and MR-PRESSO packages in R software (V 4.1.3). This study follows the STROBE-MR Statement.

## 3. Results

### 3.1. The Genetic Dataset

The sample size and download links for the GWAS summary data used in this study are provided in Table 1 and Table S1. The GSCAN comprised approximately 2.6 × 10^6^ individuals of European ancestry from 59 cohorts. This index of LifeSmk integrates smoking status, age at initiation and cessation, daily cigarette consumption, and a half-life parameter reflecting health risks such as lung cancer and all-cause mortality. The largest GWAS sourced by the NMR comprised 5185 current smokers of European ancestry, defined as a serum cotinine level of ≥10 ng/mL, an indicator of recent smoking. The FinnGen consortium (release R10, December 2023), a large-scale genomic project that combines genetic data from more than 500,000 Finnish participants with longitudinal national health registry records, provided the source of data for sarcoidosis, including 4399 cases and 405,620 controls.

### 3.2. MR Study Design

In this MR study, single-nucleotide polymorphisms (SNPs) were used as instrumental variables (IVs), satisfying the following core assumptions: (1) a strong association with smoking-related exposures; (2) independence from potential confounders; and (3) influence on sarcoidosis only through smoking, without acting via other pathways. Accordingly, SNPs included as IVs for UVMR were required to meet the following criteria: (1) genome-wide significance (*p* < 5 × 10^−8^); (2) linkage disequilibrium (LD) independence (r^2^ < 0.001 within a 10,000 kb window); (3) no obvious risk of reverse causality proved by MR Steiger filtering; (4) minor allele frequency (MAF) > 0.01; (5) F-statistic > 10, calculated using the formula (beta/SE)^2^.

The MVMR genetic instruments were selected as outlined in Figure S1. Briefly, genome-wide significant SNPs (*p* < 5 × 10^−8^) for CigDay and NMR were extracted and underwent separate within-exposure LD clumping (1000 Genomes EUR reference, r^2^ < 0.001, 10,000 kb). The resulting instruments were pooled, and a second round of cross-exposure LD clumping was performed to remove residual LD between variants from different exposures. Variants were then harmonized against sarcoidosis summary statistics; palindromic SNPs, missing variants and variants with ambiguous strand alignment were discarded. Conditional F-statistics were estimated for each exposure, and all satisfied the threshold > 10, leaving the final instrument set for primary MVMR analysis. A post-hoc exploratory MVMR-IVW sensitivity test applying MR-Steiger filtering was performed to assess instrument-filtering influence.

**Figure 1:**
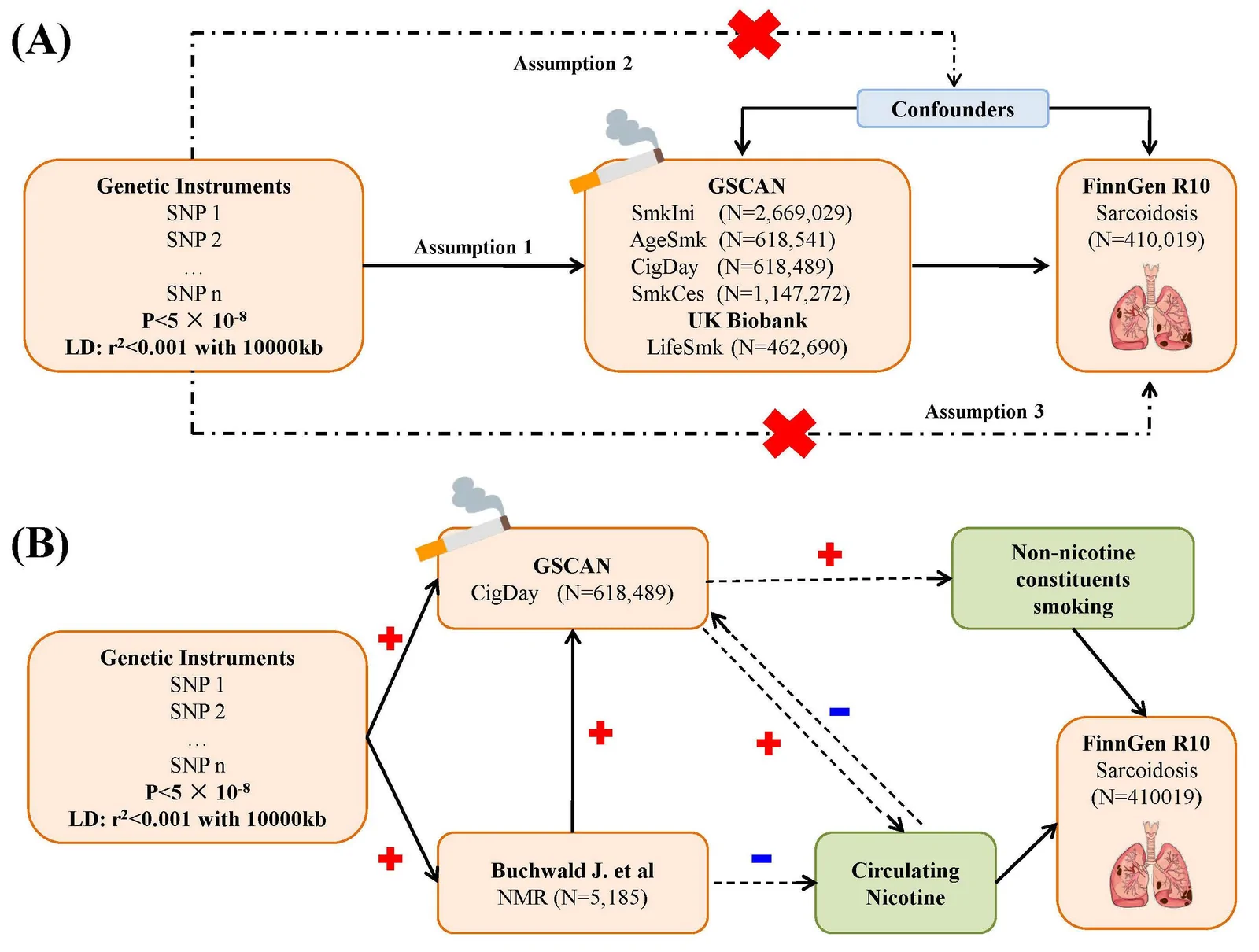
Study design. (**A**) UVMR; (**B**) MVMR. Assumption 1: IVs exhibit robust links to tobacco consumption. Assumption 2: These variables remain independent from potential confounding elements. Assumption 3: The genetic instruments influence sarcoidosis susceptibility exclusively through smoking pathways. The red cross signifies no correlation. The plus sign represents the positive correlation and the minus sign represents the negative correlation as validated in prior publications.

The study design is illustrated in Figure 1. MVMR allows the independent association of nicotine metabolism to be estimated after accounting for cigarette consumption. A higher NMR indicates that under identical nicotine exposure, the human body eliminates nicotine more rapidly. In the MVMR framework, for a given level of cigarette consumption, an individual with a higher NMR would therefore be expected to have lower circulating nicotine concentrations compared with an individual with a lower NMR, but NMR itself is a marker of metabolic rate rather than a direct measure of systemic nicotine exposure. To explore whether the inverse association between smoking and sarcoidosis may involve nicotine-related mechanisms, we analyzed the effects of five smoking-related traits on sarcoidosis using UVMR.

### 3.3. Associations Between Nicotine Metabolism-Related Traits and Sarcoidosis

**Figure 2:**
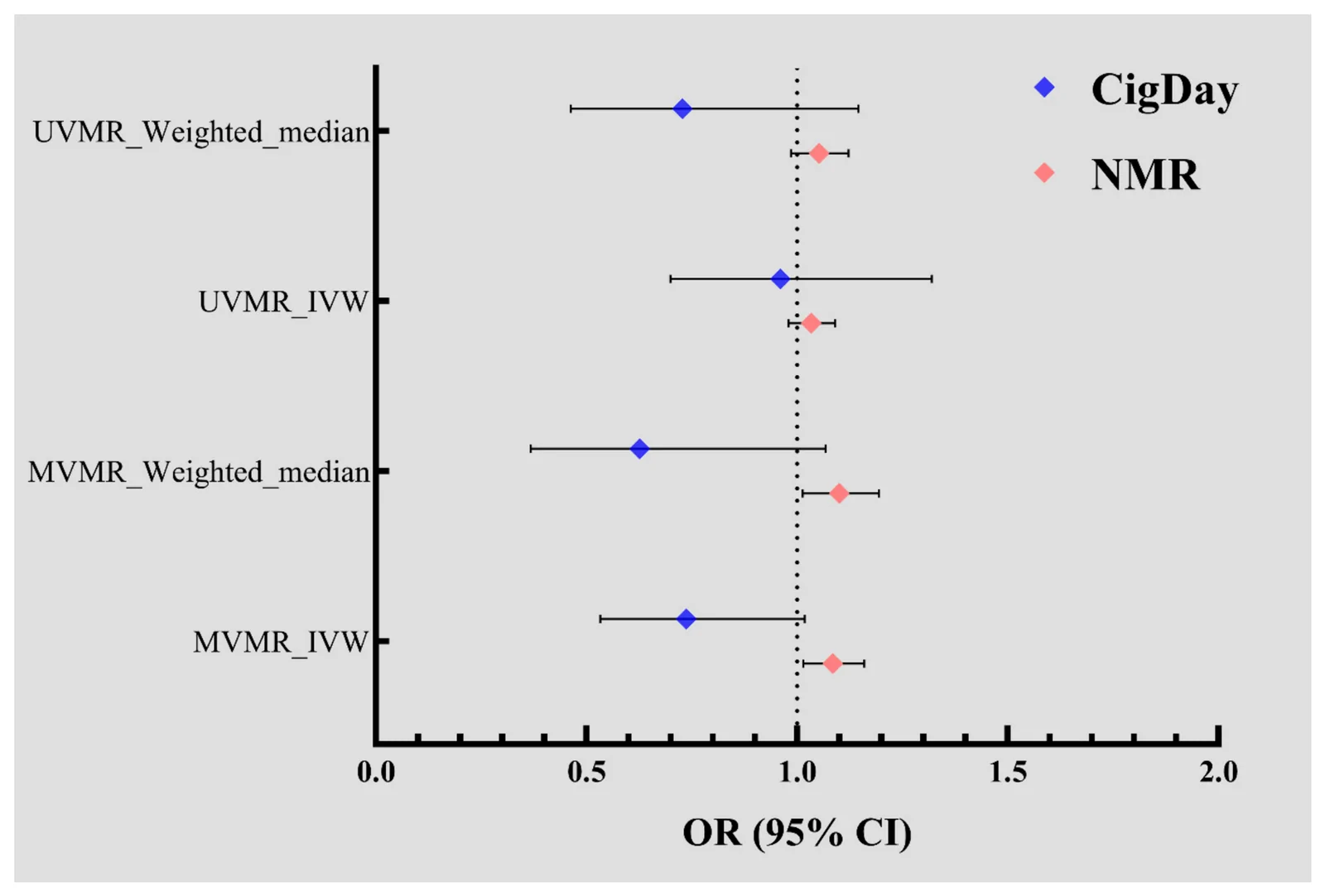
UVMR and MVMR results regarding the influence of CigDay and NMR on sarcoidosis risk.

As shown in Figure 2 (Table S2), the MVMR-IVW indicated that higher NMR was associated with higher sarcoidosis risk after adjusting for CigDay (OR = 1.08, 95% CI: 1.01 to 1.16, *p* = 0.018). No significant association was observed between CigDay and sarcoidosis risk in either UVMR or MVMR analyses. These findings were further supported by the MVMR weighted median and MVMR LASSO method (Figure S2 and Table S2). However, the MVMR-Egger intercept was 0.016 (*p* = 0.025), suggesting weak directional horizontal pleiotropy. The CigDay estimate strengthened in the MVMR-Egger model (OR = 0.43, 95% CI: 0.25–0.76, *p* = 0.004). Table S3 shows the 45 SNPs utilized in MVMR. The NMR was instrumented by six genome-wide significant variants including rs2316205, rs71358950, rs147893869, rs73042681, rs62117431 and rs34638591 (Table S4). Five of these variants are located on chromosome 19q13.2 within the CYP2A/B gene cluster region containing CYP2A6, and only one variant (rs34638591) is located on chromosome 4. The conditional F-statistics for CigDay and NMR in the MVMR analysis were 45.68 and 22.75, respectively. The Q-statistic (43.34) indicated no substantial heterogeneity (*p* = 0.41). In addition, the null association of height as a negative-control exposure argues against broad population stratification between the two GWAS as an explanation for the NMR finding (Table S5). A post-hoc sensitivity MVMR-IVW analysis, in which genetic instruments were MR-Steiger-filtered before model fitting, yielded an attenuated nominally non-significant estimate for genetically predicted NMR on sarcoidosis risk (OR = 1.07, 95% CI: 0.99–1.15, *p* = 0.081) (Table S6). Leave-one-out analysis showed broadly consistent effect directions across all iterations (Figure S3). Omitting rs72738704 attenuated the primary estimate to nominal non-significance which indicates the statistical significance of the main finding partly depends on this variant. The SNP-level scatter plot revealed no evidence of proportionality violations or outlier variants (Figure S4). Post-hoc power calculation suggested that the statistical power for the primary NMR-sarcoidosis causal estimate in MVMR was 63.5% at a two-sided α = 0.05.

### 3.4. Associations Between Smoking and Sarcoidosis

**Figure 3:**
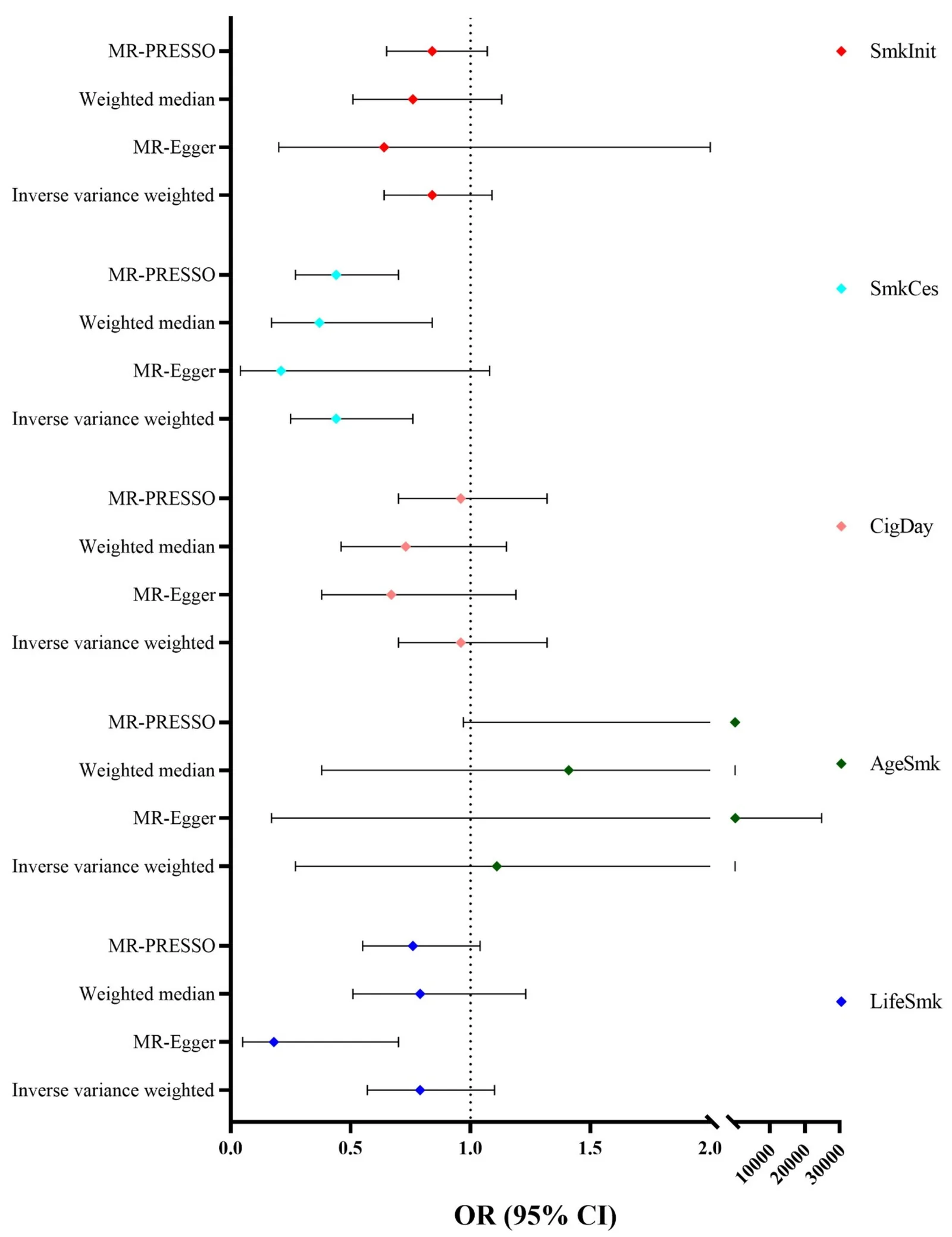
The association of genetically predicted smoking with sarcoidosis.

Table S7 lists all harmonized instrumental variables (IVs) for each risk factor-sarcoidosis pair in UVMR. We used 44, 118, 216, 10, and 21 SNPs as IVs for CigDay, LifeSmk, SmkIni, AgeSmk, and SmkCes, respectively. No statistically significant causal associations (*p* < 0.01) were observed between SmkIni (OR = 0.84, 95% CI: 0.64–1.09, *p* = 0.184), AgeSmk (OR = 1.11, 95% CI: 0.27–4.60, *p* = 0.888), CigDay (OR = 0.96, 95% CI: 0.70–1.32, *p* = 0.802), LifeSmk (OR = 0.79, 95% CI: 0.57–1.10, *p* = 0.157) and sarcoidosis. Using the IVW method, current smokers showed a reduced risk of sarcoidosis compared to former smokers (OR = 0.44, 95% CI: 0.25–0.76, *p* = 0.003) (Table 2, Figure 3). The sensitivity analyses found null associations of CurSmk (current vs. never smokers), PastSmk (former vs. never smokers) (Table S8), nicotine metabolite levels (COT and COT+3H) with sarcoidosis (Table S9).

Sensitivity analyses using the MR-Egger and weighted median methods yielded similar results (Table 2, Figure 3). Outliers were detected only for LifeSmk (rs6779302) and AgeSmk (rs12575642) in their associations with sarcoidosis by MR-PRESSO. After removing these outliers, MR-PRESSO estimates remained consistent with the primary IVW results. The F statistics in UVMR analyses ranged from 18.58 to 967.21, supporting the robustness of the causal inference (Table S7). Figures S5–S9 confirmed that no single SNP unduly influenced the overall causal estimates. With α set at 0.01, the association between SmkCes and sarcoidosis showed 89% statistical power.

As shown in Table S10, the I^2^ values for the five smoking-related traits ranged from 0% to 22.88%, suggesting low heterogeneity; this result was corroborated by Cochran’s Q test. MR-Egger intercept analysis indicated no significant pleiotropy for AgeSmk, CigDay, SmkCes, or SmkIni, except for LifeSmk. MR-PRESSO provided weak evidence of pleiotropy for LifeSmk and AgeSmk. After outlier removal, MR-Egger still suggested mild pleiotropy for AgeSmk and LifeSmk (*p* = 0.047). Tables S11 and S12 showed the heterogeneity and pleiotropy test results for the association of CurSmk, PastSmk, and nicotine metabolite with sarcoidosis. Finally, MRSD analysis confirmed the correct causal direction from smoking behavior to sarcoidosis.

## 4. Discussion

Our MVMR-IVW results showed that genetically predicted higher NMR was associated with higher sarcoidosis risk after adjusting for CigDay. UVMR analysis observed an association between the genetic liability to being a current smoker (vs. former smoker) and lower sarcoidosis risk, whereas current versus never smokers, former versus never smokers, and the nicotine metabolites COT and COT+3H are reported as null. SmkIni, AgeSmk, CigDay and LifeSmk are also not significant at the prespecified Bonferroni threshold. There is substantial heterogeneity in the findings across exposure definitions.

Smoking, a modifiable behavioral risk factor, adversely impacts multiple respiratory conditions such as lung cancer and chronic obstructive pulmonary disease [24]. However, in the case of sarcoidosis, researchers hypothesized that nicotine—rather than smoking—may exert a protective influence and conducted nicotine-patch RCTs with small sample size. The 12-week RCT reported immunological changes without clear clinical benefit, while the longer study reported changes in FVC and fatigue, but neither provides definitive evidence for nicotine treatment in sarcoidosis [8,9]. The current MR analysis is useful for generating a mechanistic hypothesis, but it can also not be translated directly into a treatment recommendation. Notably, null results from serum COT and COT+3H sensitivity analyses undermined confidence in the observed MVMR results and introduced considerable uncertainty into causal interpretation. While direct measures of circulating nicotine (e.g., blood nicotine) would be preferable, to our knowledge, no such GWAS data are currently available.

Cross-sectional studies have consistently reported a lower prevalence of smoking among individuals with sarcoidosis compared to both non-cases [25,26,27,28,29] and the general population [25,26]. Furthermore, several prospective studies have also suggested that smoking is associated with a reduced risk of developing sarcoidosis [5,30]. A Swedish study found that current smokers had a lower risk of sarcoidosis compared to never-smokers (OR = 0.48; 95% CI: 0.32–0.71), while former smokers showed a non-significant trend toward increased risk (OR = 1.33; 95% CI: 0.98–1.81) [22]. In contrast, two studies involving American veterans reported a higher risk of sarcoidosis associated with smoking. Jani et al. observed an 89% increased risk among ever-smokers [31], while Seedahmed et al. reported a 36% elevated risk among past smokers, both compared to never-smokers [32]. Similarly, the present study found that current smokers had a lower risk of sarcoidosis than former smokers, but null associations were found for CurSmk (current smokers vs. never smokers) and PastSmk (former smokers vs. never smokers). This apparent inconsistency casts doubt on a nicotine-specific protective mechanism. If higher systemic nicotine exposure genuinely protects against sarcoidosis, current smokers would be expected to have lower risk than never smokers, yet this comparison was null. Smoking persistence or cessation is a complex phenotype and cannot be equated to systemic nicotine exposure; genetic predisposition to remain a current smoker may reflect behavioural, dependence-related or other biological traits unconnected to nicotine levels. Cessation may also be prompted by pre-existing health conditions, complicating clinical interpretation. While MR-Steiger supports association directionality, it cannot resolve these interpretive ambiguities. Both current-versus-never and former-versus-never comparisons yielded null results. This pattern generates a speculative hypothesis: the significant current-versus-former-smoker association may arise largely from characteristics of the former-smoking group, rather than indicating genuine protection among current smokers. Differences in smoking duration, cumulative exposure, cessation behavior, pre-existing health status and potential reverse-causal effects may all underpin this finding.

CigDay is not a pure proxy for non-nicotine tobacco constituents, as increasing cigarette consumption also increases nicotine intake. The interpretation of the MVMR estimate depends on the chosen model structure. In the present analysis, NMR and CigDay were included as two correlated exposures, and the NMR coefficient represents the effect of NMR conditional on CigDay. This parameter can be interpreted as the effect of faster nicotine metabolism at a fixed level of cigarette consumption, but it is not equivalent to the effect of nicotine itself. Alternative specifications could change the interpretation. For example, if CigDay were omitted, the NMR estimate would combine the effect of nicotine metabolism with the effect of smoking intensity. If NMR were replaced by cotinine or cotinine+3H, the estimate would capture a different dimension of nicotine intake and metabolism. If a direct measure of blood nicotine were available, the estimate would be closer to the pharmacological effect of circulating nicotine, although such a measure would still not eliminate pleiotropy from nicotine-metabolizing enzymes. Therefore, the present findings should be viewed as model-dependent and hypothesis-generating, and future studies should compare multiple MR specifications, including bidirectional and negative-control designs, to assess the robustness of the nicotine-related interpretation.

NMR is a validated marker of cytochrome P450 family 2 subfamily A member 6 (CYP2A6)-mediated nicotine metabolic activity [12]. Under comparable nicotine intake, individuals with higher NMR metabolize nicotine more rapidly and therefore achieve lower circulating nicotine concentrations. This proxy relationship is supported by the known physiology of nicotine disposition, but it is an approximation rather than a direct measure of systemic nicotine exposure since circulating nicotine levels also depend on smoking topography, inhalation depth, and individual clearance rates that are not fully captured by the ratio alone. Consequently, the observed association between genetically predicted NMR and sarcoidosis should be interpreted as evidence regarding nicotine metabolism, and is consistent with—but does not directly establish—a protective causal effect of circulating nicotine.

Emerging research indicates that nicotine significantly modulates immune responses, particularly by attenuating Th1-mediated immune reactions through its interaction with the α7 nicotinic acetylcholine receptor (α7nAChR) [33]. The α7nAChR plays a critical role in several autoimmune disorders, largely by affecting immune regulatory mechanisms such as the inhibitory function of regulatory T (Treg) cells [34]. Activation of α7nAChR has been shown to enhance Treg activity [34] while concurrently suppressing Th1-mediated responses [33,35,36]. Thus, nicotine-mediated inhibition of Th1 immunity may underlie its possible effects in non-communicable granulomatous diseases such as sarcoidosis. However, this study provides the genetic evidence for a mechanistic hypothesis, not clinical support for nicotine therapy. It is important to emphasize that nicotine is addictive and may have off-target adverse effects, and reaffirm that smoking cessation remains strongly advised for all individuals, including those with sarcoidosis.

The present findings provide a possible genetic explanation for the inverse smoking-sarcoidosis association. MVMR represents a viable alternative in research settings where RCTs are ethically or logistically challenging—for example, when studying the long-term health effects of nicotine alone, which would require exposing non-smokers to nicotine over many years. In MR studies involving dynamic exposures such as smoking intensity, the findings reflect the effect of altering exposure susceptibility at specific time points [37,38]. Unlike RCTs, MR relies on the assumption that genetic variants exert a consistent and proportional influence on exposure levels throughout an individual’s lifetime. Thus, MR-derived estimates reflect inferred lifelong associations related to nicotine-metabolic genetic predisposition [39,40].

The restriction of the NMR GWAS to current smokers constitutes the most important limitation of the present study. The sarcoidosis outcome was derived from the general FinnGen population and therefore includes individuals with different smoking histories, whereas the MVMR assumes that the genetic effects on NMR are comparable between the two populations. This assumption may not hold if the genetic determinants of nicotine metabolism differ between persistent smokers and the general population. Restricting the exposure dataset to current smokers may introduce collider bias if genetic variants for NMR also influence the probability of smoking persistence. Under one theoretical scenario, genetic variants associated with higher NMR could distort SNP-NMR effect sizes within the selected current-smoker sample. Given that MVMR estimates depend on the ratio of outcome to exposure genetic effects, such distortion could alter the resulting causal estimate. We stress that this remains only a theoretical scenario. The real-world direction and magnitude of collider bias cannot be reliably quantified or inferred from the present data. Height was selected as a negative-control exposure because it is a highly polygenic trait with no established biological relevance to sarcoidosis (also confirmed by UVMR-IVW in this study) [22], shows a null genetic correlation with smoking status in European-ancestry populations [23], and has consistent ancestral matching with the primary exposures and outcome. The null association of height with sarcoidosis argues against broad population stratification between the two GWAS as an explanation for the NMR finding. Consequently, given its null genetic correlation with smoking, height cannot assess smoking-related selection or collider bias specific to the current-smoker restriction applied in the NMR GWAS. In addition, no single negative-control analysis can comprehensively rule out residual population stratification, horizontal pleiotropy, sample-selection effects, or the exposure-population mismatch itself. The NMR-sarcoidosis estimate should therefore be interpreted with these substantial caveats in mind, and future instruments derived from GWAS datasets covering participants with mixed smoking status will be required to fully address this source of uncertainty.

Second, while the conditional F-statistic of the NMR instruments (22.75) exceeds the conventional threshold for strong instruments (>10), this threshold alone does not preclude weak-instrument bias. The NMR GWAS comprised only 5185 current smokers, which restricts genetic architecture coverage, resulting in limited statistical power (63.5%). In the MVMR setting, weak instruments can bias causal estimates in unpredictable directions and distort the adjustment for the correlated exposure(s), thereby compromising the stability and reliability of the primary MVMR estimate, with the low statistical power further amplifying the uncertainty surrounding this estimate. Likewise, the metabolite-based sensitivity analyses were limited by the cotinine GWAS being based on only three SNPs. In addition, when MR-Steiger filtering, a reverse-causality quality-control tool for UVMR, was used as a pre-filter for MVMR instruments, the NMR-sarcoidosis association became non-significant. This highlights modest supporting evidence for the primary association and its sensitivity to instrument-filtering choices. Therefore, the NMR analysis, although supportive of the hypothesis, should therefore be regarded as exploratory and hypothesis-generating. We note, however, that this is the largest GWAS dataset currently available on NMR, and replication in a substantially larger GWAS of nicotine metabolism is required when such data become available.

Third, since five of the six NMR instruments reside within the chromosome 19 CYP2A6 region, the present MVMR analysis for NMR effectively relies on a single genetic locus. This locus-level dependence limits the scope of genetic evidence for NMR and is directly relevant to the interpretation of horizontal pleiotropy, since CYP2A6 variation influences smoking behaviours and other phenotypes in addition to nicotine metabolism. The lone chromosome 4 variant (rs34638591) provides only limited independent replication, and the NMR findings should therefore be interpreted with appropriate caution. Furthermore, genetic variants influencing NMR, particularly those in or near CYP2A6, may also affect the metabolism of other endogenous and exogenous substrates beyond nicotine, including nitrosamines, certain drugs, and possibly endogenous neuro-active compounds [41]. Such effects could create horizontal pleiotropy, whereby the instrument influences sarcoidosis risk through pathways that do not pass through circulating nicotine. Although we applied MR-Egger, weighted median, MR-PRESSO, and multivariable adjustment for CigDay, these methods cannot fully exclude pleiotropy mediated by CYP2A6-dependent pathways. In addition, CYP2A6 variants may correlate with smoking behavior, nicotine dependence, and cotinine levels, further complicating the separation of nicotine-specific effects from broader metabolic and behavioral pathways. Therefore, the possibility that the observed NMR-sarcoidosis association reflects CYP2A6-related pleiotropy rather than a direct nicotine effect should be explicitly acknowledged. Future studies using cis-MR, colocalization, and functional assays of CYP2A6 substrates may help distinguish these possibilities.

Fourth, the significant MVMR-Egger intercept (*p* = 0.025) suggests directional horizontal pleiotropy among genetic instruments, so that MVMR-Egger outputs cannot corroborate the primary MVMR-IVW estimate. Notably, CigDay was statistically significant only in the MVMR-Egger model (OR = 0.43, 95% CI: 0.25–0.76, *p* = 0.004), while no comparable signal emerged from other MVMR approaches. This cross-model discrepancy likely originates from directional pleiotropy rather than a true effect of non-nicotine tobacco constituents and adds further uncertainty to causal inference. The UVMR-Egger intercept analysis suggested the pleiotropy for LifeSmk (*p* = 0.047), which was addressed via MR-PRESSO (outlier removal: rs6779302). Although the trait LifeSmk was not significantly associated with sarcoidosis, and the pleiotropy for this trait does not impact our core findings, the presence of pleiotropy may reduce the validity of the IVs for that trait.

Fifth, the NMR GWAS meta-analysis draws in part on participants from three Finnish population-based cohorts (FinnTwin, Young Finns Study, FINRISK 2007), whose bio-samples may partially be represented in the FinnGen consortium. Given that only summary statistics are publicly available and no shared individual-level identifiers exist, the magnitude of potential sample overlap cannot be quantified. In-principle, unrecognized sample overlap could introduce bias into two-sample MR estimates, and this source of uncertainty should be kept in mind when interpreting our NMR-sarcoidosis findings. Sixth, an association with smoking or nicotine might not be identical across acute and chronic phenotypes or across pulmonary and extrapulmonary disease since sarcoidosis is clinically heterogeneous, and the FinnGen phenotype likely combines patients with different disease presentations and courses. Seventh, the current MR framework does not allow for investigation of the biological pathways mediating the observed associations.

## 5. Conclusions

The exploratory findings suggest that genetically predicted faster nicotine metabolism is associated with higher risk of sarcoidosis, but require replication and do not establish a direct protective effect of nicotine. These results should not be interpreted as justification for smoking or as evidence of clinical benefit from nicotine. Smoking cessation remains strongly advised. Future studies should explore the immune regulatory pathways through which nicotine metabolism may influence sarcoidosis pathogenesis.

## Figures and Tables

**Table 1:** The genetic dataset involved in this research. All URLs were accessed on 5 October 2026.

Trait	Population	Source	Year	N	PMID	URL
SmkIni	ever smokers (current or former) vs. never smokers	GSCAN	2022	2,669,029	36477530	https://conservancy.umn.edu/items/91f6a003-6af2-4809-9785-53dc579dc788
AgeSmk	ever smokers (current or former)	GSCAN	2022	618,541	36477530	https://conservancy.umn.edu/items/91f6a003-6af2-4809-9785-53dc579dc788
CigDay	ever smokers (current or former)	GSCAN	2022	618,489	36477530	https://conservancy.umn.edu/items/91f6a003-6af2-4809-9785-53dc579dc788
SmkCes	current smokers vs. former smokers	GSCAN	2022	1,147,272	36477530	https://conservancy.umn.edu/items/91f6a003-6af2-4809-9785-53dc579dc788
LifeSmk	never or former or current smokers	UK Biobank	2019	462,690	31689377	https://data.bris.ac.uk/data/dataset/10i96zb8gm0j81yz0q6ztei23d
NMR	current smokers	Buchwald J. et al.	2020	5185	32157176	http://ftp.ebi.ac.uk/pub/databases/gwas/summary_statistics/GCST009001-GCST010000/GCST009971
Sarcoidosis	never or former or current smokers	FinnGen R10	2024	410,019	36653562	https://storage.googleapis.com/finngen-public-data-r10/summary_stats/finngen_R10_D3_SARCOIDOSIS.gz

**Table 2:** The association of genetically predicted smoking with sarcoidosis.

Exposure	Method	nIV	*p*	OR (95% CI)
LifeSmk	Inverse variance weighted	118	1.57 × 10^−1^	0.79 (0.57, 1.10)
	MR-Egger	118	1.43 × 10^−2^	0.18 (0.05, 0.70)
	Weighted median	118	3.03 × 10^−1^	0.79 (0.51, 1.23)
	MR-PRESSO *	117	9.09 × 10^−2^	0.76 (0.55, 1.04)
AgeSmk	Inverse variance weighted	10	8.88 × 10^−1^	1.11 (0.27, 4.60)
	MR-Egger	10	2.08 × 10^−1^	64.1 (0.17, 24838.79)
	Weighted median	10	6.12 × 10^−1^	1.41 (0.38, 5.24)
	MR-PRESSO *	9	9.80 × 10^−2^	2.05 (0.97, 4.34)
CigDay	Inverse variance weighted	44	8.02 × 10^−1^	0.96 (0.70, 1.32)
	MR-Egger	44	1.75 × 10^−1^	0.67 (0.38, 1.19)
	Weighted median	44	1.70 × 10^−1^	0.73 (0.46, 1.15)
	MR-PRESSO †	44	8.04 × 10^−1^	0.96 (0.70, 1.32)
SmkCes	**Inverse variance weighted**	**21**	**3.45 × 10^−3^**	**0.44 (0.25, 0.76)**
	MR-Egger	21	7.72 × 10^−2^	0.21 (0.04, 1.08)
	Weighted median	21	1.68 × 10^−2^	0.37 (0.17, 0.84)
	**MR-PRESSO †**	**21**	**2.72 × 10^−3^**	**0.44 (0.27, 0.70)**
SmkInit	Inverse variance weighted	216	1.84 × 10^−1^	0.84 (0.64, 1.09)
	MR-Egger	216	4.41 × 10^−1^	0.64 (0.20, 2.00)
	Weighted median	216	1.74 × 10^−1^	0.76 (0.51, 1.13)
	MR-PRESSO †	216	1.51 × 10^−1^	0.84 (0.65, 1.07)

nIV, the number of SNP used; * removing one outlier; † No outlier detected.

## Data Availability

The original contributions presented in this study are included in the article/Supplementary Materials. Further inquiries can be directed to the corresponding author.

## Supplementary Materials

The following supporting information can be downloaded at: https://www.mdpi.com/article/10.3390/biomedicines14102346/s1. References [11,42] are cited in supplementary materials.

## Abbreviations

daily cigarette consumption, CigDay; nicotine metabolite ratio, NMR; univariable mendelian randomization, UVMR; Multivariable MR, MVMR; T-helper 1, Th1; regulatory T cells, Tregs; Toll-like receptors, TLR; forced vital capacity, FVC; randomized controlled trials, RCTs; Sequencing Consortium of Alcohol and Nicotine use, GSCAN; lifetime smoking index, LifeSmk; 3′-hydroxycotinine, 3H to cotinine, COT; inverse variance weighted, IVW; odds ratio, OR; confidence interval, CI; mendelian randomization steiger directionality test, MRSD; current tobacco smoking, CurSmk; past tobacco smoking, PastSmk; negative control exposure, NCE; smoking initiation, SmkIni; age of smoking initiation, AgeSmk; smoking cessation, SmkCes; single-nucleotide polymorphisms, SNPs; instrumental variables, IVs; linkage disequilibrium, LD; minor allele frequency, MAF; instrumental variables, IVs; α7 nicotinic acetylcholine receptor, α7nAChR.
