# Supplementary material for: Exploratory Mendelian Randomization Provides Hypothesis-Generating Evidence for a Possible Nicotine-Related Mechanism in Sarcoidosis
Source: Biomedicines. 2026 Oct 15;14(10):2346. doi: 10.3390/biomedicines14102346 (PMC13702295; doi:10.3390/biomedicines14102346)
Supplement: Supplementary file 1 [file biomedicines-14-02346-s001.zip › biomedicines-4548911-supplementary.pdf]

Supplementary Material

Material 1. Definition of traits (1) (2)

Smoking Initiation (SmkInit)-Binary phenotype

1. **Binary phenotype** with any participant reporting ever being a regular smoker in their life (current or former) coded “2”, while any participant who reported never being a regular smoker in their life coded “1”.

2. Does not include information about pipes/cigar/chew, or other non-cigarette forms of tobacco use.

3. This phenotype was measured in a variety of ways.

a. Have you smoked over 100 cigarettes over the course of your life?

b. Have you ever smoked every day for at least a month?

c. Have you ever smoked regularly?

Age of Initiation of Regular Smoking (AgeSmk)-continuous phenotype

1. Age (in years) at which an individual started smoking cigarettes regularly

2. Does not include information about pipes/cigars/chew, or other non-cigarette forms of tobacco use.

3. Measured in a variety of ways:

a. At what age did you begin smoking regularly?

b. How long have you smoked? Combined with What is your current age?

Cigarettes per Day (CigDay)-continuous phenotype

1. Defined as the average number of cigarettes smoked per day, either as a current smoker or former smoker. Individuals who either never smoked, or for whom there is no available data (e.g., someone was a former smoker, but for whom former smoking was never assessed) were set to missing.

2. For studies that collected a quantitative measure of cigarettes per day, where the respondent is free to provide any integer (e.g., 13 cigarettes per day) responses were binned as follows.

a. 1 = 1–5

b. 2 = 6–15

c. 3 = 16–25

d. 4 = 26–35

e. 5 = 36+

3. For studies with pre-defined bins, the pre-defined bins were used.

4. Does not include information about pipes/cigars/chew, or other non-cigarette forms of tobacco use.

5. Cigarettes per day was measured with a single question for most contributing studies using, forexample:

a. How many cigarettes do you smoke per day?

b. How many cigarettes did you smoke per day?

Smoking Cessation (SmkCes)-Binary phenotype

1. Binary phenotype with current smokers coded as "2", former smokers coded as "1", and never smokers coded as missing.

2. Does not include information about pipes/cigars/chew, or other non-cigarette forms of tobacco use.

3. Usually measured through a combination of questions, including:

a. Do you currently smoke? And have you ever smoked regularly?

b. Do you smoke? And have you smoked over 100 cigarettes in your entire life?

Lifetime smoking index-continuous phenotype

1. Core inputs (UK-Biobank self-report, one-time baseline questionnaire)

a. Smoking status: never / former / current

b. Age started, age stopped (if former)

c. Cigarettes per day (CPD) – capped at 150; hand-rolled tobacco converted 1 g ≈ 1 cigarette

2. Pack-years style quantity + decay term

a. For every year of smoking the contribution = CPD ÷ 20 × 1 year.

b. This contribution is then discounted backward in time with an exponential decay (half-life τ = 18 years).

c. The same 18-year half-life was chosen by simulating lung-cancer and all-cause-mortality models in UK-Biobank and taking the best-fitting value.

3. Final index value

a. Sum the discounted pack-years over all smoking-years → continuous score.

b. Never-smokers are set to 0.

c. One SD of the score corresponds to ≈ "20 cigs/day for 15 years then quit 17 y ago" or "60 cigs/day for 13 y then quit 22 y ago".

The GWAS of this score (N=462,690 Europeans) yielded 126 independent genome-wide SNPs that were used as the MR instrument for "lifetime smoking".

References

1. Ye T, Shao J, H K. Debiased Inverse-Variance Weighted Estimator in Two-Sample Summary-Data Mendelian Randomization. Institute of Mathematical Statistics. 2021;4.

2. Wootton RE, Richmond RC, Stuijfzand BG, Lawn RB, Sallis HM, Taylor GMJ, et al. Evidence for causal effects of lifetime smoking on risk for depression and schizophrenia: a Mendelian randomisation study. Psychol Med. 2020;50(14):2435-43.

Material 2. The R for power calculation of MVMR

```
# Post-hoc power calculation: NMR-sarcoidosis (MVMR, two-sided alpha=0.05)
beta_nmr <- 0.08113248
se_nmr <- 0.03439547
alpha <- 0.05
k <- 45
p <- 2
df <- k - p
delta_nmr <- abs(beta_nmr) / se_nmr
t_critical <- qt(1 - alpha/2, df)
power_nmr <- 1 - pt(t_critical, df, ncp = delta_nmr) + pt(-t_critical, df, ncp = delta_nmr)
cat("Power for NMR-sarcoidosis =", round(power_nmr*100, 1), "%\n")
```

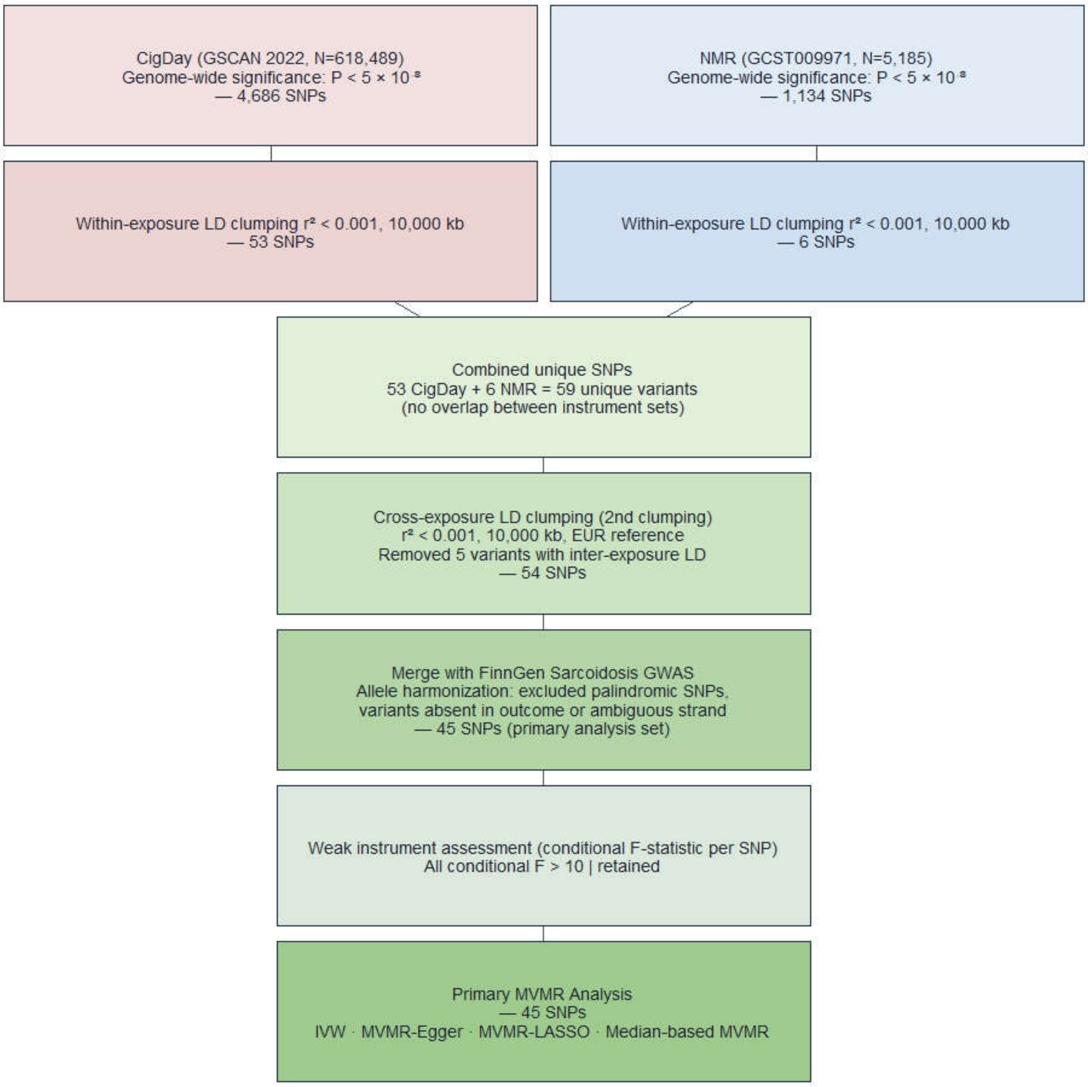

Figure S1. Instrument selection flow diagram of SNPs used in MVMR

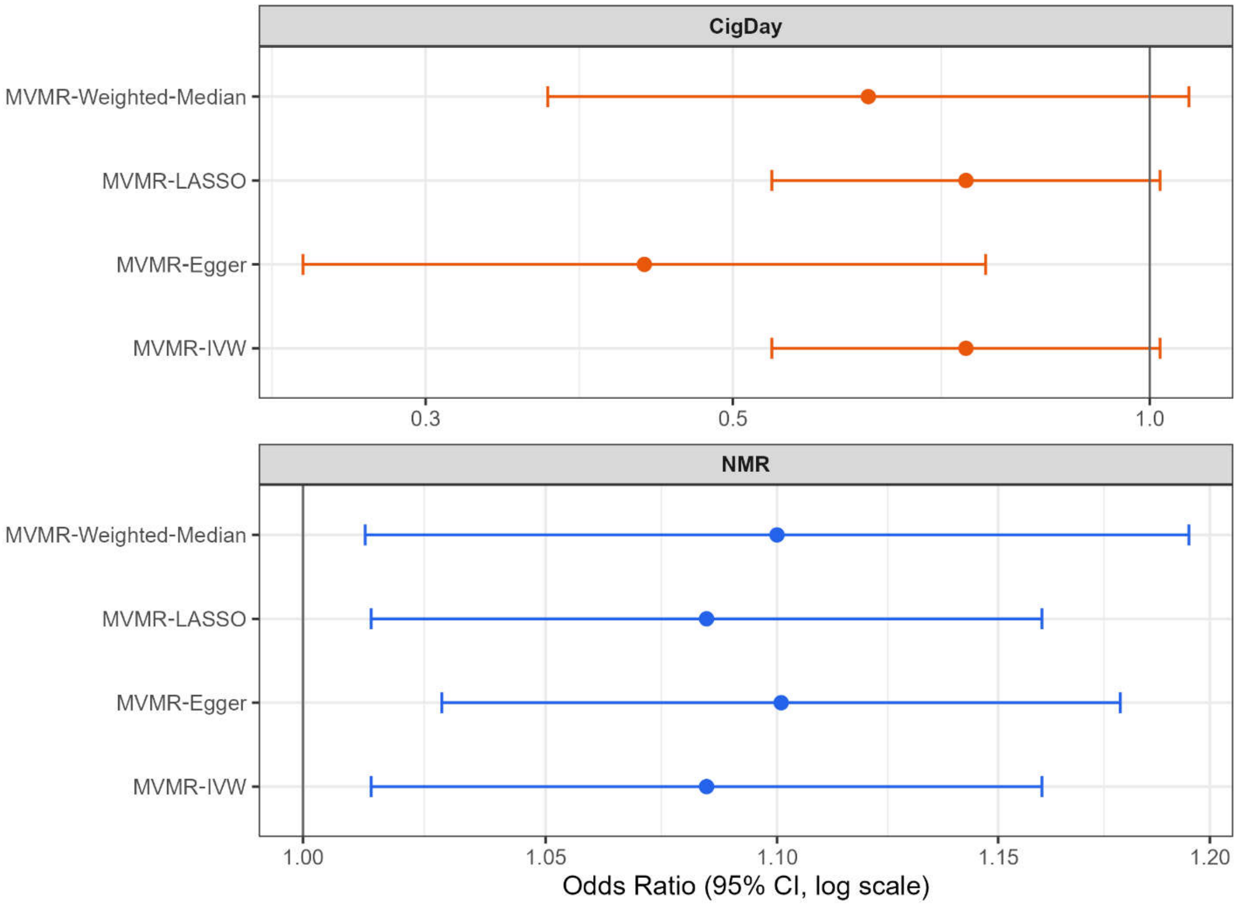

Figure S2. Sensitivity analysis of MVMR estimates for CigDay and NMR on sarcoidosis

Odds ratio with 95% confidence intervals (CI) (log scale) derived from MVMR-IVW, MVMR-Egger, MVMR-LASSO, and weighted-median MVMR using the 45-SNP instrument set.

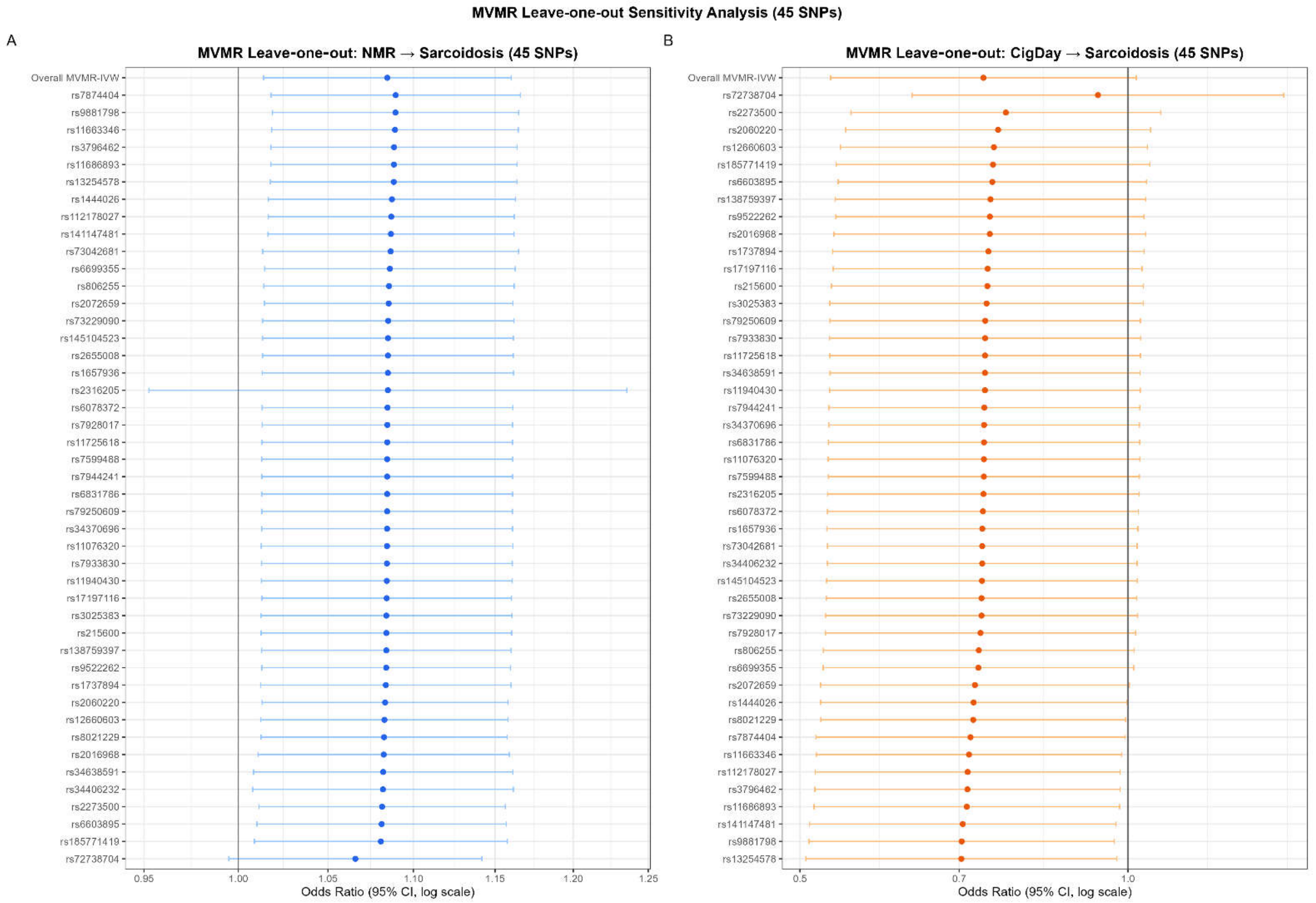

Figure S3. Leave-one-out analysis for MVMR. Each horizontal line represents the MVMR-IVW estimate after omitting one single SNP; the bottom row shows the overall MVMR-IVW estimate using all included genetic variants. Odds ratios and 95% confidence intervals (CI) are plotted on a logarithmic scale.

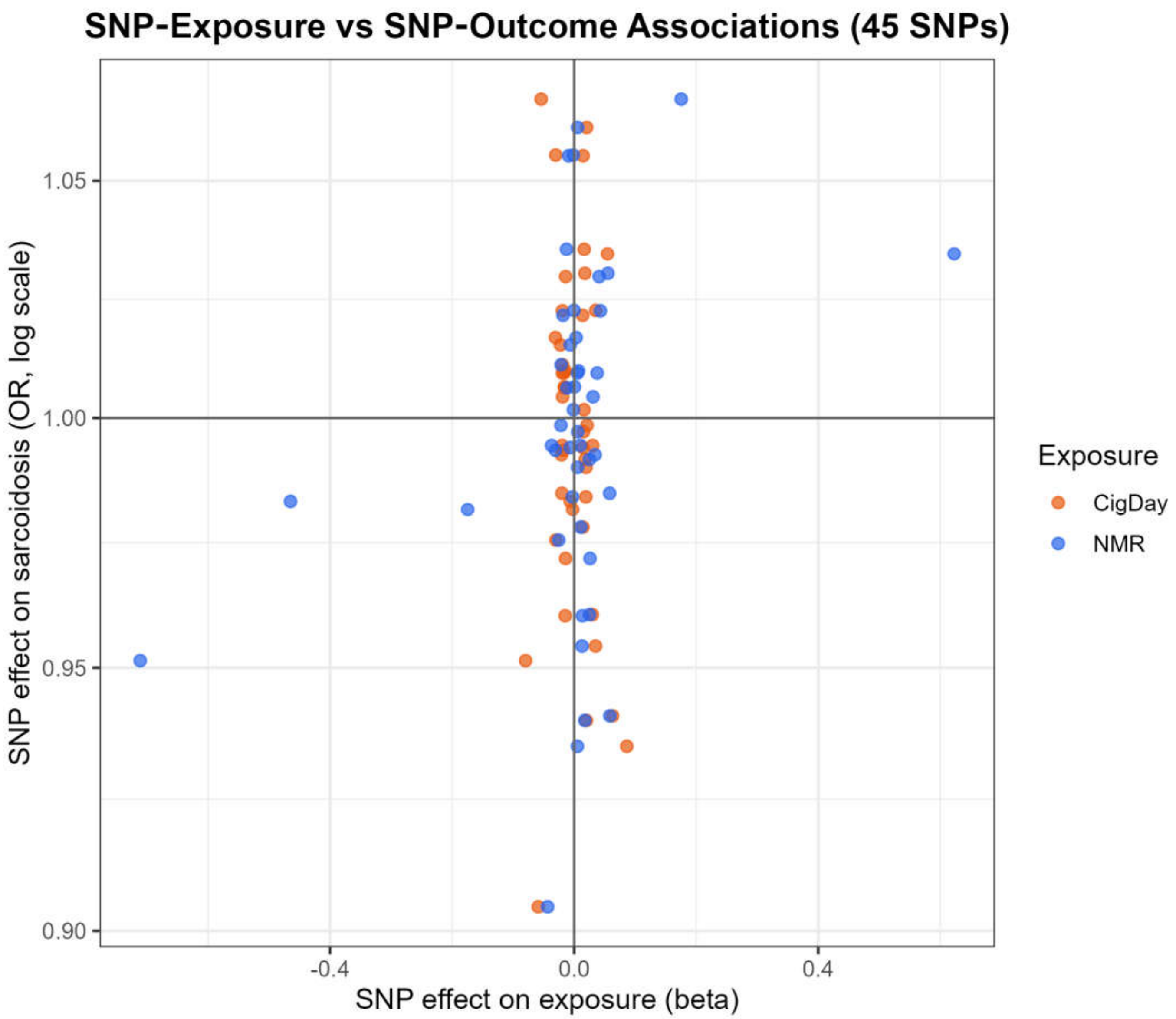

Figure S4. Scatter plot of SNP-exposure associations versus SNP-outcome associations. Each point represents a genetic variant. Orange points show the association between each SNP and CigDay; blue points show the association between each SNP and NMR. The horizontal axis denotes the SNP effect on the corresponding exposure (beta coefficient). The vertical axis denotes the SNP effect on sarcoidosis, expressed as odds ratio (OR) on a logarithmic scale. The vertical solid line at beta = 0 indicates no genetic association with the exposure; the horizontal solid line at OR = 1 indicates no genetic association with sarcoidosis. A total of 45 SNPs were included in this plot.

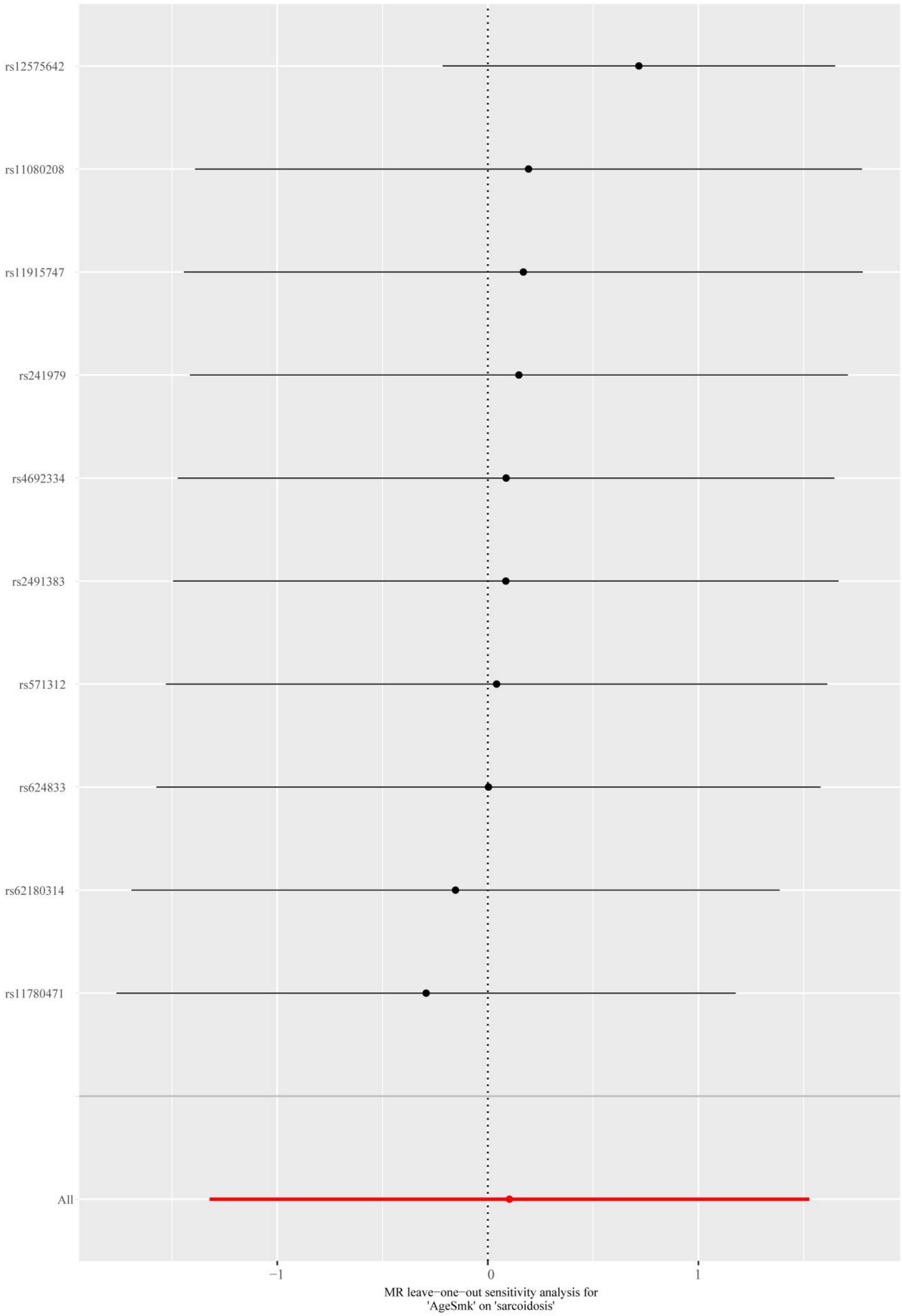

Figure S5. The leave-one-out plot of the effect of single SNP of AgeSmk on sarcoidosis.

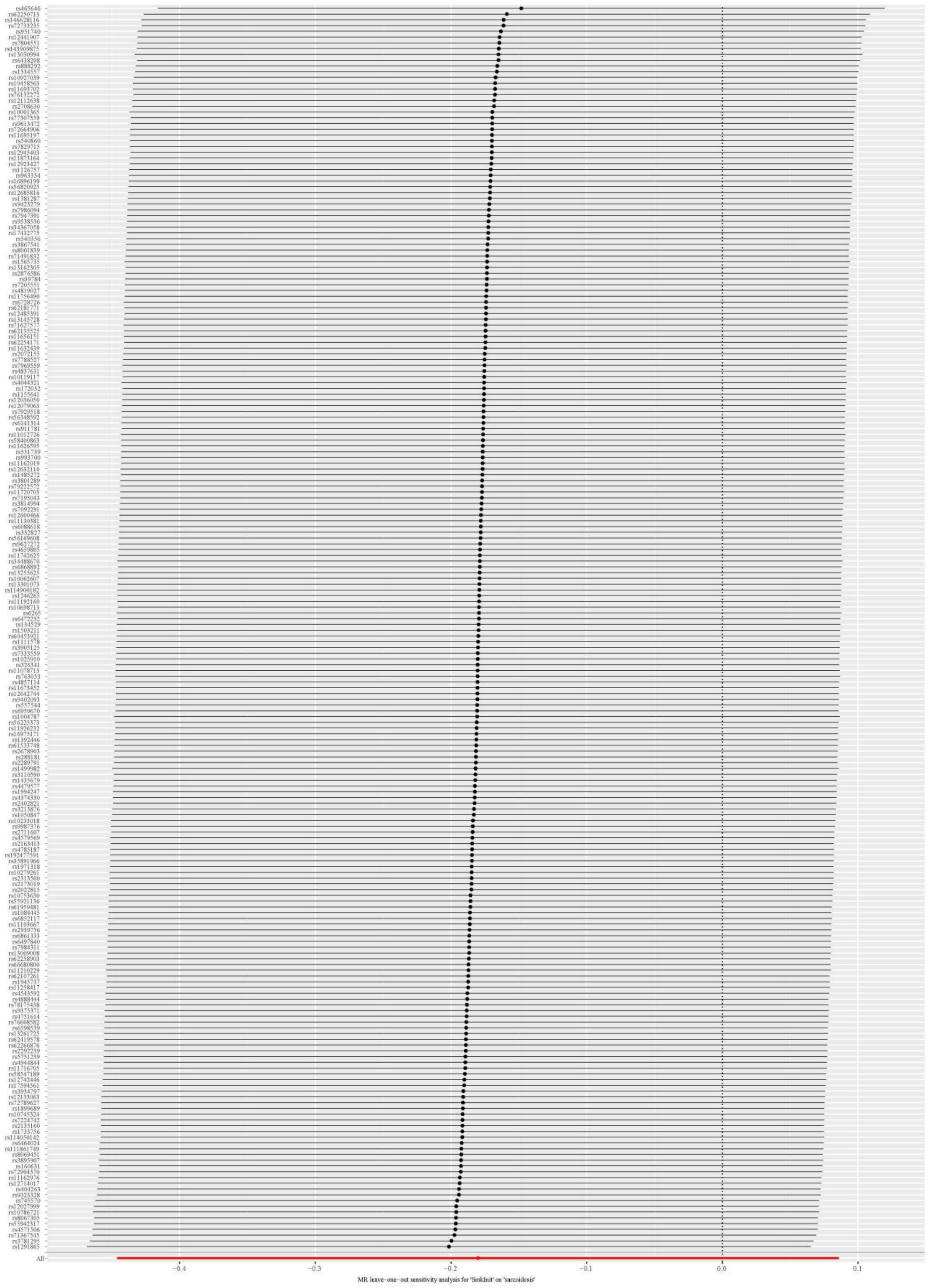

Figure S6. The leave-one-out plot of the effect of single SNP of SmkIni on sarcoidosis.

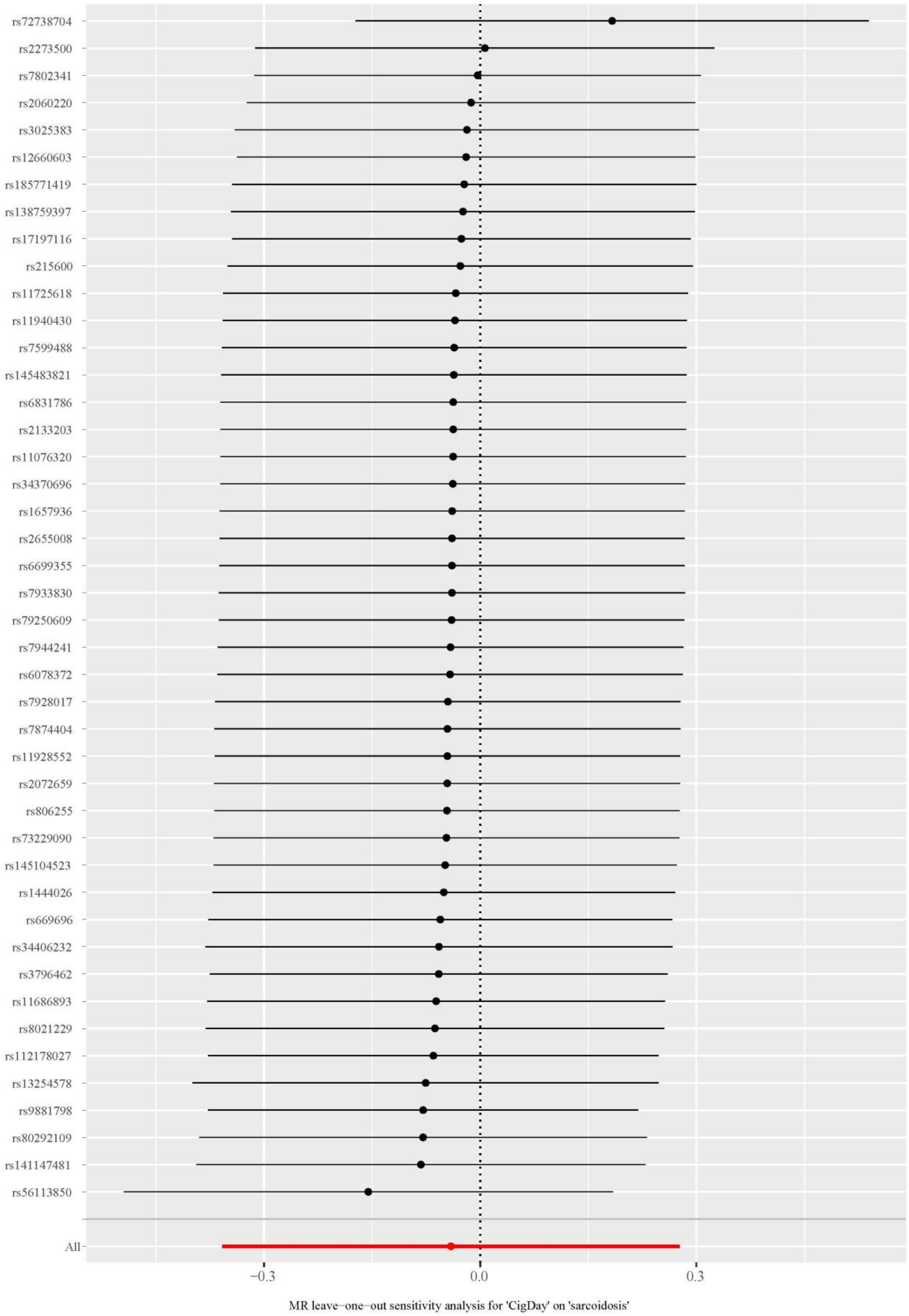

Figure S7. The leave-one-out plot of the effect of single SNP of CigDay on sarcoidosis.

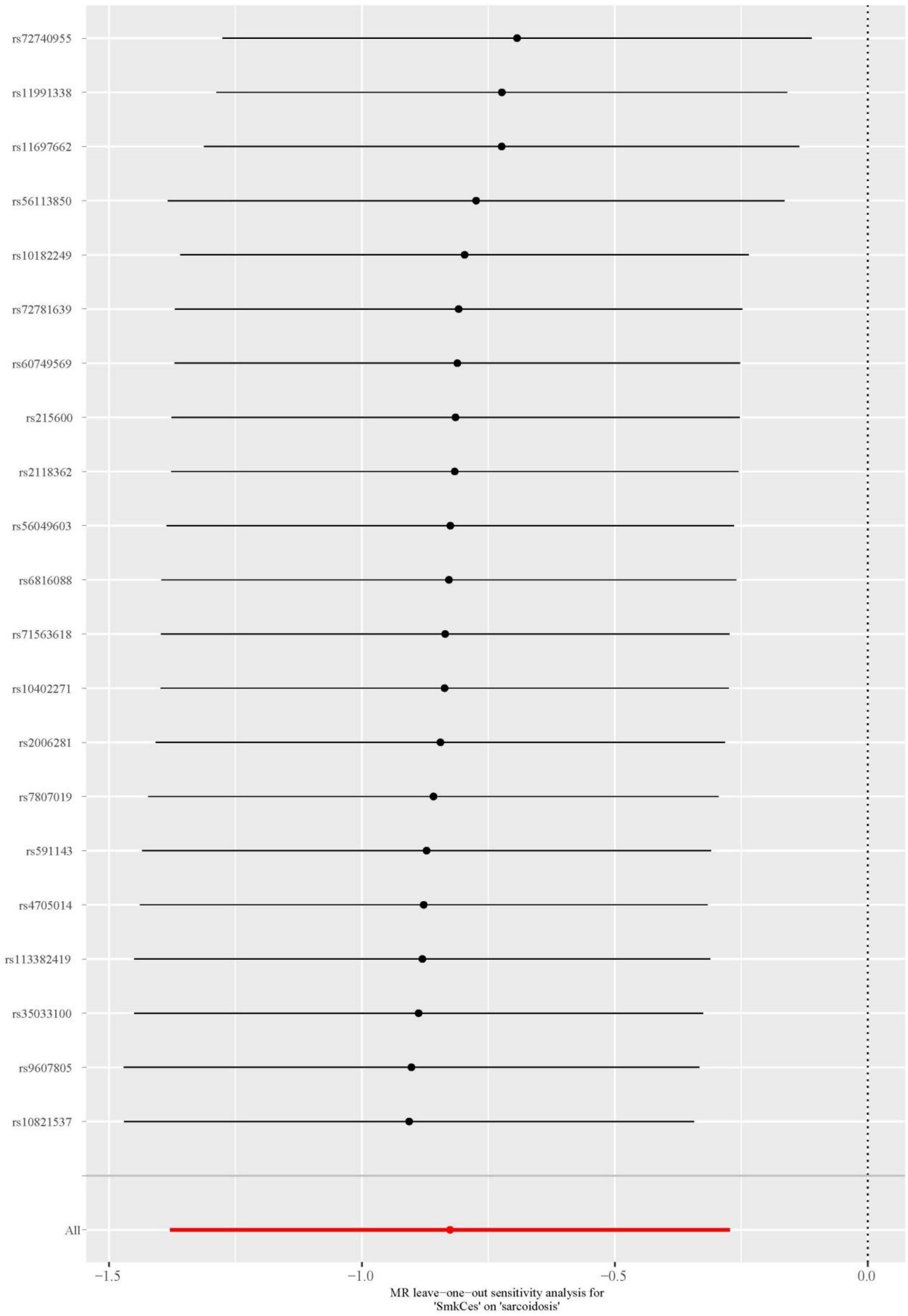

Figure S8. The leave-one-out plot of the effect of single SNP of SmkCes on sarcoidosis.

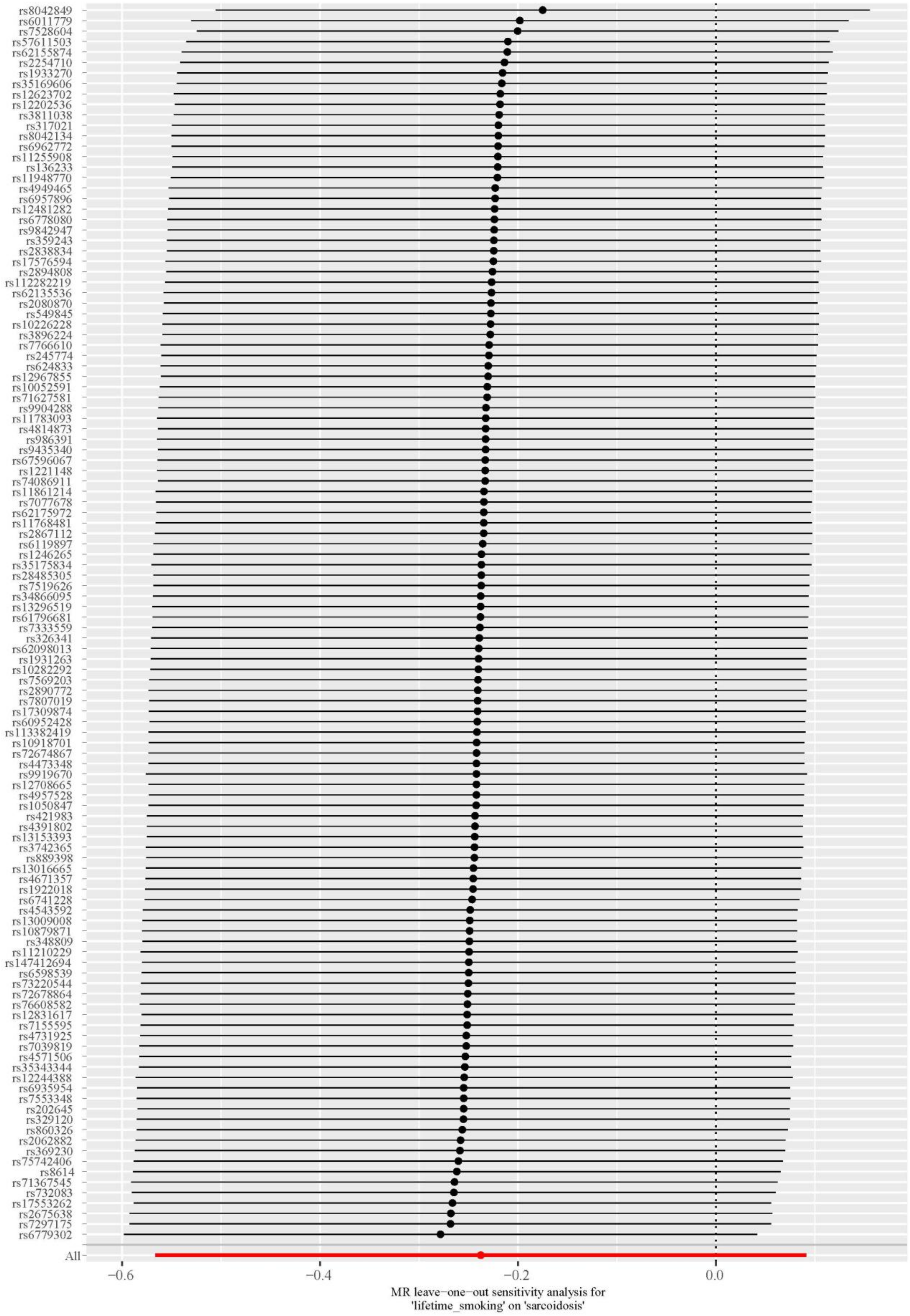

Figure S9. The leave-one-out plot of the effect of single SNP of LifeSmk on sarcoidosis.

Table S1. The genetic dataset involved in this research

Trait	Population	Source	Year	N	PMID	URL
Current tobacco smoking	current smokers vs. never smokers	UKBiobank	2018	462,434	30349118	https://opengwas.io/datasets/ukb-b-223#
Past tobacco smoking	former smokers vs. never smokers	UKBiobank	2018	424,960	30349118	https://opengwas.io/datasets/ukb-b-2134
Cotinine levels	current smokers	Buchwald J. et al	2020	5,185	32157176	http://ftp.ebi.ac.uk/pub/databases/gwas/summary_statistics/GCST009001-GCST010000/GCST009965
Cotinine and 3'-hydroxycotinine levels	current smokers	Buchwald J. et al	2020	5,185	32157176	http://ftp.ebi.ac.uk/pub/databases/gwas/summary_statistics/GCST009001-GCST010000/GCST009970
Height (Negative Control Exposure)	general population	Loh PR. et al	2018	673,878	29892013	https://opengwas.io/datasets/ebi-a-GCST90029008

Table S2. Sensitivity analyses of MVMR estimate for CigDay and NMR on sarcoidosis

Method	Exposure	OR (95% CI)	*P*_exact
MVMR-IVW	CigDay	0.74 (0.53, 1.02)	0.0636
MVMR-IVW	NMR	1.08 (1.01, 1.16)	0.0183
MVMR-Egger	CigDay	0.43 (0.24, 0.76)	0.0037
MVMR-Egger	NMR	1.10 (1.03, 1.18)	0.0057
MVMR-LASSO	CigDay	0.74 (0.53, 1.02)	0.0636
MVMR-LASSO	NMR	1.08 (1.01, 1.16)	0.0183
MVMR-Weighted-Median	CigDay	0.63 (0.37, 1.07)	0.0854
MVMR-Weighted-Median	NMR	1.10 (1.01, 1.19)	0.0241

Table S3. The detailed information of used SNP in multivariable mendelian randomization.

SNP	betaYG	sebetaYG	betaX1	betaX2	sebetaX1	sebetaX2
rs11076320	0.00925209	0.0242945	-0.0164	0.037784	0.003	0.020787
rs112178027	0.0597527	0.0326653	0.0205	0.005599	0.003	0.02749
rs11663346	-0.0288871	0.0224362	-0.0141	0.026016	0.003	0.019845
rs11686893	0.0347292	0.0234024	0.0165	-0.012493	0.003	0.021044
rs11725618	-0.00848274	0.0253149	0.0181	0.024967	0.003	0.022562
rs11940430	0.00969579	0.0243859	-0.0146	0.007066	0.003	0.02123
rs12660603	0.0540789	0.0407725	-0.0303	-0.001521	0.006	0.049148
rs13254578	0.0221	0.0264367	0.0353	-0.000248	0.003	0.024024
rs138759397	-0.0611471	0.0705749	0.0628	0.05851	0.009	0.065449
rs141147481	-0.100407	0.0631004	-0.0588	-0.043496	0.009	0.058589
rs1444026	0.0210877	0.0231765	0.014	-0.018149	0.002	0.019971
rs145104523	-0.00148624	0.0365416	0.0212	-0.021624	0.004	0.030653
rs1657936	0.00435551	0.0311942	-0.0189	0.030959	0.003	0.025587
rs17197116	-0.0404039	0.0530524	0.0298	0.025089	0.005	0.04207
rs1737894	-0.0161876	0.0225675	0.0192	-0.002743	0.003	0.020342
rs185771419	0.0655554	0.0568203	-0.054	0.175458	0.007	0.076496
rs2016968	0.0220189	0.0240307	-0.0193	0.043021	0.003	0.020937
rs2060220	-0.062098	0.0306588	0.0201	0.017417	0.004	0.027475
rs2072659	-0.0251178	0.0371504	-0.0301	-0.025345	0.004	0.03472
rs215600	0.0150278	0.0249359	-0.0225	-0.006431	0.003	0.021358
rs2273500	-0.0468375	0.0272918	0.035	0.012892	0.003	0.025081
rs2316205	0.0337603	0.0228234	0.0547	0.622835	0.002	0.019538
rs2655008	0.00171142	0.0270607	0.0161	-0.001208	0.003	0.023373
rs3025383	0.0165239	0.0297342	-0.0307	0.003227	0.003	0.026269
rs34370696	0.00923164	0.0334725	-0.0192	0.005446	0.003	0.027563
rs34406232	-0.0498456	0.0719029	-0.0796	-0.711165	0.007	0.063751
rs34638591	-0.0188166	0.0225062	-0.00241	-0.17454	0.003	0.02457
rs3796462	-0.0406325	0.0241084	-0.0147	0.013554	0.003	0.021408
rs6078372	-0.00280909	0.0226471	0.0153	0.005714	0.003	0.019857
rs6603895	0.0290505	0.0230322	-0.014	0.041003	0.002	0.020066
rs6699355	-0.00755229	0.0314413	-0.0207	0.034273	0.004	0.028385
rs6831786	0.00641023	0.0224593	-0.0161	0.000711	0.003	0.023461
rs72738704	-0.0673795	0.0243028	0.0863	0.005332	0.003	0.021133
rs73042681	-0.0171163	0.0759075	-0.00643	-0.464859	0.008	0.084931
rs73229090	-0.00564599	0.0385986	0.0304	-0.036898	0.004	0.032218
rs7599488	-0.00604585	0.022503	0.0144	-0.006585	0.002	0.019645
rs7874404	-0.0154299	0.0253779	-0.02	0.058197	0.003	0.041323
rs79250609	0.0109414	0.0290753	-0.0189	-0.021447	0.003	0.026873
rs7928017	-0.00660894	0.0253062	-0.0176	-0.030466	0.002	0.021057
rs7933830	-0.0101229	0.0262323	0.0194	0.005483	0.003	0.022029
rs7944241	0.00619544	0.0226445	-0.0156	-0.011579	0.003	0.019803
rs8021229	0.0297183	0.0227893	0.0176	0.055325	0.003	0.020389
rs806255	-0.00563626	0.026589	-0.0197	0.009946	0.003	0.022982
rs9522262	-0.0224576	0.0225945	0.0144	0.010723	0.002	0.019906
rs9881798	0.0539299	0.0231411	0.0147	-0.009034	0.003	0.020069

Table S4. The six genome-wide significant variants used to instrument the nicotine metabolite ratio (NMR) in the MVMR analysis.

SNP	Effect allele	Other allele	EAF	Beta	SE	P	Chr	Position (GRCh37)	Nearest gene	CYP2A6 region
rs2316205	C	T	0.50834	0.622835	0.01954	1e-200	19	41155000	CYP2A7/CYP2A6	Yes
rs71358950	A	G	0.23312	0.179436	0.02348	2.22e-14	19	41267000	CYP2A cluster (intergenic)	Yes
rs147893869	G	C	0.02289	-0.854539	0.06633	6.12e-38	19	41129000	Near EGLN2	Yes
rs73042681	C	G	0.02099	-0.464859	0.08493	4.52e-08	19	40514000	MAP3K10	Yes
rs62117431	G	A	0.07232	0.364033	0.05583	7.26e-11	19	42230000	ARHGEF1	Yes
rs34638591	C	T	0.57009	-0.174540	0.02457	1.26e-12	4	69300000	TMPRSS11E	No

Table S5. The association of genetically predicted NCE with sarcoidosis

Exposure	Method	*P*	OR (95% CI)
NCE in UVMR	Inverse variance weighted	3.55E-01	0.96 (0.88, 1.05)
F>=23.89	MR-Egger	2.92E-01	1.10 (0.92, 1.30)
nSNP=797	Weighted median	8.62E-01	1.01 (0.88, 1.16)
	MR-PRESSO *	4.96E-01	0.98 (0.90, 1.06)
NCE in MVMR	Inverse variance weighted	9.19E-01	0.97 (0.50, 1.86)
F>=20.08	Weighted median	7.46E-01	0.95 (0.68, 1.32)

* removing rs11681299, rs3008892 and rs9268652

NCE, height was used as a negative control exposure in this study

UVMR: I^2: 18.86%, heterogeneity Q_pval: 0.00002295627, egger_intercept: -0.003665805, MR Egger P: 0.07580626, MR PRESSO P: 0.905

Table S6. The comparison of the MVMR results before and after steiger filtering test

MVMR IVW Model	CigDay OR (95% CI)	CigDay P-value	NMR OR (95% CI)	NMR P-value	CigDay F	NMR F	Q_Pleio_P
45 SNPs	0.737 (0.533-1.017)	0.0636	1.085 (1.014–1.160)	0.0183	45.7	22.8	0.414
39 SNPs*	0.892 (0.584-1.362)	0.598	1.067 (0.992–1.147)	0.0811	31.3	21.9	0.614

*Steiger-filtered rs112178027, rs12660603, rs13254578, rs34638591, rs6831786, rs72738704

Table S7. The detailed information of used SNP in univariable mendelian randomization in primary analysis.

exposure trait	SNP	nearest_genes	effect allele	other allele	beta.exposure	beta.outcome	se.exposure	se.outcome	eaf.exposure	eaf.outcome	pval.exposure	pval.outcome	MAF	F-value	id.outcome
AgeSmk	rs11080208	ASIC2	C	T	-0.0176	0.0119577	0.003	0.0247507	0.752485	0.745836	8.63E-10	0.629006	0.254164	34.42	sarcoidosis
AgeSmk	rs11780471	EPHX2	A	G	0.0369	0.0886366	0.005	0.0436183	0.0755467	0.0658123	1.59E-12	0.0421444	0.0658123	54.46	sarcoidosis
AgeSmk	rs11915747	CADM2	G	C	0.0174	-0.00609054	0.003	0.022107	0.367793	0.391475	1.75E-11	0.78293	0.391475	33.64	sarcoidosis
AgeSmk	rs12575642	FERMT3	T	G	-0.0183	0.10791	0.003	0.0271939	0.183897	0.180467	1.69E-08	7.24E-05	0.180467	37.21	sarcoidosis
AgeSmk	rs241979	CRACR2A	A	G	-0.0159	0.00777938	0.003	0.027174	0.246521	0.193364	3.87E-08	0.774663	0.193364	28.09	sarcoidosis
AgeSmk	rs2491383	SORCS3	G	T	0.0152	0.0043391	0.003	0.023417	0.61829	0.693786	1.30E-08	0.852996	0.306214	25.67	sarcoidosis
AgeSmk	rs4692334	STIM2	C	G	0.018	0.00620039	0.003	0.0327755	0.803181	0.876923	4.05E-08	0.849954	0.123077	36	sarcoidosis
AgeSmk	rs571312	MC4R	A	C	0.0176	0.0136781	0.003	0.0276634	0.239563	0.18502	2.21E-09	0.620989	0.18502	34.42	sarcoidosis
AgeSmk	rs62180314	WDPCP	C	G	0.0236	0.0437799	0.003	0.0299275	0.207753	0.15384	1.39E-14	0.143505	0.15384	61.88	sarcoidosis
AgeSmk	rs624833	ADD1	G	T	0.0158	0.0157988	0.003	0.0226244	0.328032	0.356558	4.98E-09	0.484985	0.356558	27.74	sarcoidosis
SmkCes	rs10182249	KLHL29	A	G	-0.0136	0.0237627	0.002	0.0220377	0.646123	0.601363	1.61E-08	0.280911	0.398637	46.24	sarcoidosis
SmkCes	rs10402271	BCAM	G	T	-0.0132	0.00603182	0.002	0.0221257	0.34493	0.388947	4.30E-08	0.785149	0.388947	43.56	sarcoidosis
SmkCes	rs10821537	DBH	G	A	-0.0153	-0.0208647	0.002	0.022891	0.652087	0.640016	1.58E-10	0.362043	0.359984	58.52	sarcoidosis
SmkCes	rs113382419	FAM163B	A	C	0.0501	0.00514128	0.004	0.0599572	0.0904573	0.0329208	4.96E-41	0.931666	0.0329208	156.88	sarcoidosis
SmkCes	rs11697662	CHRNA4	T	C	-0.0301	0.0483966	0.003	0.0250059	0.804175	0.751022	2.26E-25	0.0529407	0.248978	100.67	sarcoidosis
SmkCes	rs11991338	TNKS	A	G	0.0182	-0.0621014	0.003	0.0263191	0.170974	0.216034	2.31E-08	0.0182966	0.216034	36.8	sarcoidosis
SmkCes	rs2006281	PPP1R13B	T	C	-0.0139	0.00373165	0.002	0.0215861	0.534791	0.472602	8.11E-10	0.862752	0.472602	48.3	sarcoidosis
SmkCes	rs2118362	SOX6	C	T	0.0153	-0.017916	0.003	0.0267628	0.286282	0.20505	2.19E-09	0.503218	0.20505	26.01	sarcoidosis
SmkCes	rs215600	PDE1C	A	G	-0.0144	0.0171192	0.002	0.0239967	0.667992	0.721025	1.50E-09	0.475599	0.278975	51.84	sarcoidosis
SmkCes	rs35033100	BHLHE23	C	T	-0.0307	-0.0341515	0.005	0.049146	0.0417495	0.0525152	2.31E-08	0.487119	0.0525152	37.7	sarcoidosis
SmkCes	rs4705014	SGCD	A	G	-0.0132	-0.0126913	0.002	0.0221412	0.661034	0.616379	1.98E-08	0.566512	0.383621	43.56	sarcoidosis
SmkCes	rs56049603	BSN	G	C	0.0159	-0.0134253	0.003	0.0273955	0.202783	0.189819	8.42E-09	0.624094	0.189819	28.09	sarcoidosis
SmkCes	rs56113850	CTC-490E21.12,CYP2A6	C	T	-0.033	0.0351755	0.002	0.0221798	0.592445	0.563463	3.85E-47	0.112757	0.436537	272.25	sarcoidosis
SmkCes	rs591143	SEMA6D	T	C	-0.0144	-0.00816019	0.002	0.0226374	0.629225	0.656066	5.00E-10	0.718494	0.343934	51.84	sarcoidosis
SmkCes	rs60749569	CHRNA6	T	A	-0.0266	0.0410826	0.004	0.0534956	0.0805169	0.0423028	5.38E-10	0.442509	0.0423028	44.22	sarcoidosis
SmkCes	rs6816088	ARAP2	C	T	0.0217	-0.017027	0.003	0.0270867	0.153082	0.197063	3.24E-10	0.529604	0.197063	52.32	sarcoidosis
SmkCes	rs71563618	MDFIC	G	A	0.0207	-0.0106081	0.004	0.0339357	0.084493	0.115085	6.91E-09	0.754588	0.115085	26.78	sarcoidosis
SmkCes	rs72740955	PSMA4	T	C	0.0252	-0.0520467	0.002	0.0228956	0.373757	0.334963	1.30E-25	0.0230128	0.334963	158.76	sarcoidosis
SmkCes	rs72781639	UBXN2A	G	C	0.0203	-0.0295208	0.003	0.0353355	0.16004	0.102178	1.12E-09	0.403469	0.102178	45.79	sarcoidosis
SmkCes	rs7807019	CTTNBP2	G	A	0.0147	0.000793648	0.002	0.0218739	0.470179	0.581183	1.00E-10	0.971057	0.418817	54.02	sarcoidosis
SmkCes	rs9607805	PHF5A	T	C	0.0189	0.00946413	0.003	0.0228656	0.702783	0.668124	8.88E-14	0.678946	0.331876	39.69	sarcoidosis
CigDay	rs11076320	TOX3	A	C	-0.0164	0.00542998	0.003	0.0233245	0.632207	0.690299	1.06E-10	0.815915	0.309701	29.88	sarcoidosis
CigDay	rs112178027	CRYBA1	T	C	0.0205	0.0567379	0.003	0.0313958	0.181909	0.137039	8.02E-10	0.0707343	0.137039	46.69	sarcoidosis
CigDay	rs11686893	ACVR2A	C	T	0.0165	0.0300801	0.003	0.0224701	0.286282	0.359695	3.28E-10	0.180677	0.359695	30.25	sarcoidosis
CigDay	rs11725618	EPHA5	C	T	0.0181	-0.0110388	0.003	0.0242045	0.313121	0.271958	5.71E-11	0.648343	0.271958	36.4	sarcoidosis
CigDay	rs11928552	IP6K2	C	T	-0.0166	-0.00696129	0.003	0.0226763	0.651093	0.65757	2.71E-09	0.758855	0.34243	30.62	sarcoidosis
CigDay	rs11940430	TSPAN5	A	T	-0.0146	0.01068	0.003	0.0234188	0.654076	0.695499	2.94E-08	0.64836	0.304501	23.68	sarcoidosis
CigDay	rs12660603	MMS22L	C	T	-0.0303	0.0529827	0.006	0.0391248	0.0437376	0.084168	4.24E-08	0.175673	0.084168	25.5	sarcoidosis
CigDay	rs13254578	CHRNB3	C	G	0.0353	0.0291064	0.003	0.0254208	0.777336	0.763496	4.81E-33	0.252216	0.236504	138.45	sarcoidosis
CigDay	rs138759397	CHRNB4	C	G	0.0628	-0.0616672	0.009	0.0677215	0.0258449	0.0270659	5.71E-13	0.362506	0.0270659	48.69	sarcoidosis
CigDay	rs141147481	PSMA4	G	C	-0.0588	-0.122145	0.009	0.0601882	0.0228628	0.0334464	3.61E-10	0.042419	0.0334464	42.68	sarcoidosis
CigDay	rs1444026	KHDRBS3	G	T	0.014	0.0166569	0.002	0.0221855	0.446322	0.407756	2.15E-08	0.452772	0.407756	49	sarcoidosis
CigDay	rs145104523	BRWD1	T	C	0.0212	0.0221294	0.004	0.0350881	0.125249	0.10514	1.09E-08	0.528249	0.10514	28.09	sarcoidosis
CigDay	rs145483821	EPHA3	G	T	-0.0165	0.00607355	0.003	0.021533	0.439364	0.514649	1.41E-08	0.777899	0.485351	30.25	sarcoidosis
CigDay	rs1657936	ZNF280D	T	C	-0.0189	0.00457149	0.003	0.029917	0.781312	0.846539	6.52E-10	0.878551	0.153461	39.69	sarcoidosis
CigDay	rs17197116	AMBRA1	C	T	0.0298	-0.0635804	0.005	0.051178	0.084493	0.0465358	4.52E-11	0.214112	0.0465358	35.52	sarcoidosis
CigDay	rs185771419	MORF4L1	T	G	-0.054	0.0509445	0.007	0.0544355	0.0238569	0.0403201	1.20E-13	0.349341	0.0403201	59.51	sarcoidosis
CigDay	rs2060220	TENM2	T	A	0.0201	-0.0594363	0.004	0.0294126	0.133201	0.162448	2.16E-08	0.0433032	0.162448	25.25	sarcoidosis
CigDay	rs2072659	CHRNB2	G	C	-0.0301	-0.00957269	0.004	0.0357287	0.101392	0.102788	6.44E-13	0.788755	0.102788	56.63	sarcoidosis
CigDay	rs2133203	AK5	T	C	-0.0162	0.00514541	0.003	0.0219857	0.450298	0.400642	2.42E-09	0.814959	0.400642	29.16	sarcoidosis
CigDay	rs215600	PDE1C	A	G	-0.0225	0.0171192	0.003	0.0239967	0.667992	0.721025	2.81E-18	0.475599	0.278975	56.25	sarcoidosis
CigDay	rs2273500	CHRNA4	C	T	0.035	-0.0451694	0.003	0.0261572	0.146123	0.217549	7.09E-24	0.0841957	0.217549	136.11	sarcoidosis
CigDay	rs2655008	STAG1	T	A	0.0161	-0.00348891	0.003	0.0259613	0.730616	0.779964	1.78E-08	0.893095	0.220036	28.8	sarcoidosis
CigDay	rs3025383	DBH	C	T	-0.0307	0.029657	0.003	0.0285934	0.180915	0.169883	6.01E-22	0.299644	0.169883	104.72	sarcoidosis
CigDay	rs34370696	ILDR2	T	C	-0.0192	0.00738481	0.003	0.0321153	0.175944	0.129109	2.36E-08	0.818133	0.129109	40.96	sarcoidosis
CigDay	rs34406232	EGLN2,RAB4B-EGLN2	A	C	-0.0796	-0.0448251	0.007	0.0690003	0.0258449	0.0256134	1.21E-26	0.515928	0.0256134	129.31	sarcoidosis
CigDay	rs3796462	UNC5C	T	C	-0.0147	-0.0297134	0.003	0.02315	0.701789	0.684447	3.11E-08	0.199312	0.315553	24.01	sarcoidosis
CigDay	rs56113850	CTC-490E21.12,CYP2A6	C	T	0.0622	0.0351755	0.002	0.0221798	0.592445	0.563463	1.36E-137	0.112757	0.436537	967.21	sarcoidosis
CigDay	rs6078372	BTBD3	A	G	0.0153	0.00136935	0.003	0.0217546	0.420477	0.43311	1.22E-09	0.94981	0.43311	26.01	sarcoidosis
CigDay	rs669696	NFAT5	A	C	-0.0194	-0.0168225	0.003	0.0217389	0.423459	0.430161	1.69E-11	0.439023	0.430161	41.82	sarcoidosis
CigDay	rs6699355	DLGAP3	T	C	-0.0207	0.00364681	0.004	0.0301926	0.877734	0.847067	3.05E-08	0.903862	0.152933	26.78	sarcoidosis
CigDay	rs6831786	CENPC	A	C	-0.0161	0.00503604	0.003	0.0215595	0.558648	0.522827	6.51E-10	0.815305	0.477173	28.8	sarcoidosis
CigDay	rs72738704	IREB2	C	G	0.0863	-0.0534242	0.003	0.0232537	0.370775	0.315043	1.00E-200	0.0215929	0.315043	827.52	sarcoidosis
CigDay	rs73229090	CLU	A	C	0.0304	0.0126486	0.004	0.0368937	0.11332	0.0943698	1.65E-14	0.73172	0.0943698	57.76	sarcoidosis
CigDay	rs7599488	BCL11A	T	C	0.0144	-0.00759845	0.002	0.0216027	0.417495	0.480519	8.42E-09	0.725037	0.480519	51.84	sarcoidosis
CigDay	rs7802341	GATSL2	A	T	-0.0223	0.0585186	0.004	0.0267078	0.746521	0.781103	1.82E-08	0.0284472	0.218897	31.08	sarcoidosis
CigDay	rs7874404	ADAMTSL2	T	C	-0.02	-0.00637616	0.003	0.0243688	0.213718	0.268369	6.64E-10	0.79359	0.268369	44.44	sarcoidosis
CigDay	rs79250609	MMAB	T	C	-0.0189	0.00247526	0.003	0.0278592	0.176938	0.185308	7.93E-09	0.929202	0.185308	39.69	sarcoidosis
CigDay	rs7928017	DRD2	A	C	-0.0176	-0.00605378	0.002	0.0240097	0.407555	0.287209	2.22E-12	0.800934	0.287209	77.44	sarcoidosis
CigDay	rs7933830	SOX6	T	C	0.0194	-0.00282769	0.003	0.0252105	0.319085	0.242359	5.23E-13	0.910694	0.242359	41.82	sarcoidosis
CigDay	rs7944241	HSD17B12	G	A	-0.0156	-0.000217378	0.003	0.0217357	0.409543	0.43338	4.59E-10	0.992021	0.43338	27.04	sarcoidosis
CigDay	rs8021229	KLC1	T	C	0.0176	0.0287988	0.003	0.0218778	0.296223	0.41374	2.14E-11	0.188058	0.41374	34.42	sarcoidosis
CigDay	rs80292109	HIST1H1D	G	A	-0.0194	-0.045545	0.003	0.0218434	0.295229	0.418998	1.24E-11	0.037063	0.418998	41.82	sarcoidosis
CigDay	rs806255	YTHDF3	A	G	-0.0197	-0.00817293	0.003	0.0255314	0.732604	0.769243	5.53E-12	0.748883	0.230757	43.12	sarcoidosis
CigDay	rs9881798	PLCL2	C	A	0.0147	0.06191	0.003	0.0220527	0.39165	0.378059	4.47E-09	0.00499494	0.378059	24.01	sarcoidosis
SmkInit	rs10001365	TTC29	A	G	-0.0152	0.0204633	0.002	0.0218252	0.378728	0.423583	4.32E-21	0.348449	0.423583	57.76	sarcoidosis
SmkInit	rs1004787	SIX3	A	G	0.0161	-0.00175188	0.002	0.022285	0.535785	0.625585	3.91E-24	0.937341	0.374415	64.8	sarcoidosis
SmkInit	rs10062607	THBS4	A	C	0.0103	-0.00475582	0.002	0.0222274	0.635189	0.624051	2.28E-10	0.830576	0.375949	26.52	sarcoidosis
SmkInit	rs10119117	LINGO2	T	C	0.00901	-0.0143171	0.002	0.0216261	0.497018	0.45051	0.000000018	0.507952	0.45051	20.3	sarcoidosis
SmkInit	rs10233018	CTTNBP2	G	A	0.0139	0.00446219	0.002	0.0219972	0.512922	0.597137	1.49E-18	0.83925	0.402863	48.3	sarcoidosis
SmkInit	rs1025910	DCC	C	G	-0.0123	0.00164573	0.002	0.0232316	0.294235	0.310881	4.83E-13	0.943525	0.310881	37.82	sarcoidosis
SmkInit	rs10279261	EXOC4	A	G	-0.0122	-0.00842045	0.002	0.0224724	0.629225	0.640644	6.87E-14	0.707882	0.359356	37.21	sarcoidosis
SmkInit	rs10458563	ELAVL4	G	A	0.0171	-0.0321713	0.002	0.0270905	0.190855	0.196828	2.17E-18	0.235011	0.196828	73.1	sarcoidosis
SmkInit	rs1050847	ZCCHC14	T	C	-0.0105	-0.00563968	0.002	0.0216524	0.550696	0.506483	3.71E-11	0.794505	0.493517	27.56	sarcoidosis
SmkInit	rs10698713	TULP4	A	G	-0.0201	0.0165508	0.004	0.0816787	0.0526839	0.0177293	1.84E-08	0.839422	0.0177293	25.25	sarcoidosis
SmkInit	rs10745324	KCND3	G	A	-0.00962	-0.0324774	0.002	0.0230912	0.672962	0.679616	1.97E-08	0.159581	0.320384	23.14	sarcoidosis
SmkInit	rs10753630	NUF2	T	C	0.0109	0.0110347	0.002	0.0217855	0.348907	0.420028	9.96E-11	0.612493	0.420028	29.7	sarcoidosis
SmkInit	rs10786721	AS3MT,BORCS7-ASMT	A	C	0.0179	0.0204513	0.002	0.0221424	0.416501	0.382928	7.91E-27	0.355679	0.382928	80.1	sarcoidosis
SmkInit	rs1084445	NEGR1	C	T	-0.0121	-0.0239128	0.002	0.0311839	0.225646	0.136089	1.80E-10	0.443182	0.136089	36.6	sarcoidosis
SmkInit	rs10927039	AKT3	C	T	-0.0127	0.0398677	0.002	0.0261125	0.196819	0.216307	2.39E-10	0.126819	0.216307	40.32	sarcoidosis
SmkInit	rs11012726	SKIDA1	C	T	0.0143	-0.0101281	0.002	0.023249	0.33002	0.31645	4.25E-17	0.6631	0.31645	51.12	sarcoidosis
SmkInit	rs11078713	CHD3	G	A	-0.0126	0.00163618	0.002	0.0216912	0.408549	0.534361	2.77E-15	0.939872	0.465639	39.69	sarcoidosis
SmkInit	rs11103667	OLFM1	T	C	0.0129	0.0193827	0.002	0.0290933	0.178926	0.163349	1.47E-10	0.505266	0.163349	41.6	sarcoidosis
SmkInit	rs1111578	KSR2	T	G	-0.0125	0.00238107	0.002	0.0274973	0.15507	0.190204	8.22E-09	0.930995	0.190204	39.06	sarcoidosis
SmkInit	rs11130381	CHDH	T	C	-0.00862	0.007449	0.002	0.0215973	0.525845	0.485473	4.83E-08	0.730167	0.485473	18.58	sarcoidosis
SmkInit	rs11162019	LMO4	T	C	-0.00971	0.0119339	0.002	0.0231665	0.379722	0.314301	3.40E-09	0.606457	0.314301	23.57	sarcoidosis
SmkInit	rs11162976	ADGRL2	C	A	0.0108	0.0443701	0.002	0.0262238	0.306163	0.213782	6.47E-10	0.0906505	0.213782	29.16	sarcoidosis
SmkInit	rs111861749	TFAP2B	A	G	-0.0179	-0.0292402	0.003	0.029553	0.124254	0.161713	3.16E-12	0.32246	0.161713	35.6	sarcoidosis
SmkInit	rs11192160	SORCS3	C	G	0.0139	-0.00514716	0.002	0.0301174	0.185885	0.151221	8.66E-10	0.8643	0.151221	48.3	sarcoidosis
SmkInit	rs11210229	LRRIQ3	G	A	-0.014	-0.0109533	0.002	0.0222414	0.564612	0.626536	4.94E-18	0.622385	0.373464	49	sarcoidosis
SmkInit	rs11258417	BEND7	T	C	-0.0101	-0.0192139	0.002	0.0231825	0.335984	0.320107	3.83E-10	0.407213	0.320107	25.5	sarcoidosis
SmkInit	rs1126757	IL11	T	C	0.0104	-0.0248942	0.002	0.0217662	0.472167	0.565364	4.15E-11	0.252745	0.434636	27.04	sarcoidosis
SmkInit	rs114050142	MED12L	A	T	-0.0227	-0.0479372	0.003	0.0434499	0.0616302	0.0668996	2.56E-11	0.269908	0.0668996	57.25	sarcoidosis
SmkInit	rs114900182	AC004158.1	G	C	-0.0209	0.00735388	0.003	0.0378542	0.0586481	0.0895223	1.45E-12	0.845965	0.0895223	48.53	sarcoidosis
SmkInit	rs1155641	SPATA8	A	G	0.0102	-0.0134858	0.002	0.0223419	0.333996	0.367156	7.72E-10	0.546104	0.367156	26.01	sarcoidosis
SmkInit	rs11626595	KIF26A	T	C	-0.0151	0.0172307	0.002	0.0338932	0.156064	0.113474	2.73E-12	0.611185	0.113474	57	sarcoidosis
SmkInit	rs11632439	ABHD17C	G	A	0.00898	-0.0170556	0.002	0.0218887	0.44831	0.425919	1.31E-08	0.435865	0.425919	20.16	sarcoidosis
SmkInit	rs11656151	MAPT	G	A	-0.0128	0.0187435	0.002	0.0265429	0.200795	0.2072	5.61E-12	0.48009	0.2072	40.96	sarcoidosis
SmkInit	rs11673452	PEPD	C	T	0.015	-0.000867716	0.003	0.0340595	0.11332	0.114922	3.40E-09	0.979675	0.114922	25	sarcoidosis
SmkInit	rs11693702	SLC4A10	A	T	0.014	-0.0270078	0.002	0.0225287	0.416501	0.354853	9.33E-19	0.230599	0.354853	49	sarcoidosis
SmkInit	rs11695197	FAHD2B	A	G	0.0138	-0.0288158	0.002	0.0256283	0.115308	0.239912	6.28E-09	0.260853	0.239912	47.61	sarcoidosis
SmkInit	rs11716705	PDCD6IP	G	A	0.0117	0.0234396	0.002	0.0242465	0.267396	0.272846	1.40E-10	0.333684	0.272846	34.22	sarcoidosis
SmkInit	rs11720703	FOXP1	T	C	0.0109	-0.00873951	0.002	0.0216347	0.506958	0.451794	5.58E-12	0.686243	0.451794	29.7	sarcoidosis
SmkInit	rs11742625	ARRDC3	C	T	0.0157	-0.00815181	0.002	0.0340236	0.115308	0.113225	2.19E-10	0.810646	0.113225	61.62	sarcoidosis
SmkInit	rs11756490	MCHR2	A	T	-0.0137	0.0257637	0.002	0.0314082	0.129225	0.135765	6.20E-09	0.412053	0.135765	46.92	sarcoidosis
SmkInit	rs11873164	SETBP1	T	C	-0.0155	0.0377411	0.002	0.0315931	0.131213	0.134319	1.63E-12	0.232243	0.134319	60.06	sarcoidosis
SmkInit	rs11926232	ERC2	G	A	0.0206	0.00645164	0.004	0.0596519	0.0467197	0.0339613	1.16E-08	0.913873	0.0339613	26.52	sarcoidosis
SmkInit	rs12027999	UBAP2L	C	T	-0.0184	-0.031537	0.002	0.0272001	0.151093	0.199473	1.54E-13	0.246274	0.199473	84.64	sarcoidosis
SmkInit	rs12036050	PTBP2	C	T	0.0115	-0.0145193	0.002	0.0248816	0.284294	0.249595	7.18E-11	0.559531	0.249595	33.06	sarcoidosis
SmkInit	rs12079063	RABGAP1L	G	A	-0.00872	0.0135138	0.002	0.0216766	0.447316	0.438156	3.88E-08	0.533005	0.438156	19.01	sarcoidosis
SmkInit	rs12112638	AUTS2	G	A	-0.0122	0.0324016	0.002	0.023977	0.234592	0.279298	8.74E-12	0.176579	0.279298	37.21	sarcoidosis
SmkInit	rs12133063	BARHL2	A	C	0.0116	0.0221472	0.002	0.0217466	0.371769	0.555505	1.15E-12	0.308479	0.444495	33.64	sarcoidosis
SmkInit	rs12441907	BNC1	A	C	-0.015	0.0472727	0.002	0.027912	0.166998	0.183727	6.09E-14	0.0903337	0.183727	56.25	sarcoidosis
SmkInit	rs1246265	SLC28A3	C	T	0.0116	-0.00440528	0.002	0.0239733	0.701789	0.71874	1.27E-11	0.854203	0.28126	33.64	sarcoidosis
SmkInit	rs12485391	SOX2	A	C	0.0138	-0.0417028	0.002	0.0413257	0.12326	0.0747094	1.89E-08	0.312915	0.0747094	47.61	sarcoidosis
SmkInit	rs12600466	CA10	T	A	0.0115	-0.00893539	0.002	0.0261637	0.301193	0.214698	1.30E-11	0.732713	0.214698	33.06	sarcoidosis
SmkInit	rs12632110	SEMA3F	G	A	-0.0138	0.00978991	0.002	0.023586	0.663022	0.703428	1.18E-16	0.678088	0.296572	47.61	sarcoidosis
SmkInit	rs12642744	STIM2	T	G	-0.0124	0.000636528	0.002	0.0281047	0.757455	0.823032	5.98E-11	0.981931	0.176968	38.44	sarcoidosis
SmkInit	rs12685816	PTPRD	A	G	0.0145	-0.0304195	0.002	0.0290483	0.150099	0.165477	1.66E-10	0.295005	0.165477	52.56	sarcoidosis
SmkInit	rs12714017	CTNNA2	C	T	0.0103	0.0315082	0.002	0.0216444	0.532803	0.444282	6.57E-10	0.14547	0.444282	26.52	sarcoidosis
SmkInit	rs12742446	PHC2	T	G	0.0119	0.0214923	0.002	0.0227255	0.630219	0.662956	2.74E-10	0.344284	0.337044	35.4	sarcoidosis
SmkInit	rs1291865	CELF2	T	G	0.0116	0.0450348	0.002	0.0217197	0.531809	0.562596	2.12E-13	0.0381294	0.437404	33.64	sarcoidosis
SmkInit	rs12923427	XYLT1	T	C	-0.0136	0.0563107	0.002	0.0371622	0.219682	0.0921941	4.96E-12	0.129704	0.0921941	46.24	sarcoidosis
SmkInit	rs12945403	RFNG	C	T	-0.00941	0.0356824	0.002	0.0241171	0.616302	0.723263	1.79E-08	0.138993	0.276737	22.14	sarcoidosis
SmkInit	rs13009008	AC013461.1	G	A	-0.0097	-0.0164896	0.002	0.0223246	0.651093	0.623841	6.92E-09	0.460133	0.376159	23.52	sarcoidosis
SmkInit	rs13030994	ZEB2	A	G	0.0207	-0.0222093	0.002	0.0218026	0.508946	0.571869	2.42E-39	0.308367	0.428131	107.12	sarcoidosis
SmkInit	rs13145728	MAML3	C	G	-0.0122	0.0150323	0.002	0.0224517	0.360835	0.361136	6.87E-14	0.503152	0.361136	37.21	sarcoidosis
SmkInit	rs13162305	CTNND2	T	A	0.00975	-0.0208662	0.002	0.0227988	0.33499	0.333284	1.15E-08	0.36007	0.333284	23.77	sarcoidosis
SmkInit	rs13255625	GPIHBP1	G	A	0.0116	-0.00546562	0.002	0.024536	0.695825	0.737581	4.03E-12	0.823723	0.262419	33.64	sarcoidosis
SmkInit	rs13261725	NECAB1	C	G	0.0131	0.0184002	0.002	0.0238786	0.261431	0.284203	3.43E-13	0.44096	0.284203	42.9	sarcoidosis
SmkInit	rs13301073	MAPKAP1	A	G	0.0109	-0.00442535	0.002	0.0223126	0.348907	0.36841	3.13E-11	0.842784	0.36841	29.7	sarcoidosis
SmkInit	rs1334557	GATA3	T	C	0.0132	-0.0553084	0.002	0.0305211	0.245527	0.145073	1.51E-12	0.0699649	0.145073	43.56	sarcoidosis
SmkInit	rs134529	TTC28	C	T	-0.0116	0.00308168	0.002	0.0229419	0.379722	0.330917	8.86E-13	0.893145	0.330917	33.64	sarcoidosis
SmkInit	rs1381287	C14orf177	T	C	0.0123	-0.0199597	0.002	0.0215773	0.50497	0.492274	7.51E-15	0.354948	0.492274	37.82	sarcoidosis
SmkInit	rs1392446	CDH6	T	C	0.00981	0.00154933	0.002	0.0215545	0.532803	0.519498	5.60E-10	0.942698	0.480502	24.06	sarcoidosis
SmkInit	rs1435679	C15orf41	A	G	0.01	0.00361087	0.002	0.0221891	0.581511	0.622763	2.86E-10	0.87073	0.377237	25	sarcoidosis
SmkInit	rs143909875	FANCL	C	T	0.0176	-0.046965	0.003	0.0305588	0.107356	0.146218	3.74E-12	0.124324	0.146218	34.42	sarcoidosis
SmkInit	rs146628116	EYS	A	T	0.0144	-0.0379317	0.002	0.0223619	0.379722	0.369054	1.43E-14	0.0898359	0.369054	51.84	sarcoidosis
SmkInit	rs1485272	SUMF1	C	T	-0.00907	0.010791	0.002	0.0218154	0.37674	0.425728	4.07E-08	0.620848	0.425728	20.57	sarcoidosis
SmkInit	rs1499982	IGSF11	T	C	0.0216	-0.000733646	0.002	0.0262735	0.835984	0.782582	3.01E-23	0.977723	0.217418	116.64	sarcoidosis
SmkInit	rs1503211	GRID2	A	G	0.00999	-0.00284339	0.002	0.0222635	0.454274	0.375048	3.43E-10	0.898374	0.375048	24.95	sarcoidosis
SmkInit	rs1565735	EPHX2	A	T	-0.0232	0.0186135	0.002	0.0305171	0.199801	0.146258	7.49E-32	0.541904	0.146258	134.56	sarcoidosis
SmkInit	rs160631	ICK	G	T	-0.0105	-0.0348893	0.002	0.0237945	0.728628	0.711598	3.56E-09	0.142574	0.288402	27.56	sarcoidosis
SmkInit	rs16896199	EYS	T	A	0.0118	-0.0316005	0.002	0.026503	0.166998	0.210985	5.61E-09	0.233128	0.210985	34.81	sarcoidosis
SmkInit	rs16975171	PIK3C3	A	C	-0.0172	-0.0037776	0.003	0.0425292	0.10835	0.0686319	5.29E-09	0.929222	0.0686319	32.87	sarcoidosis
SmkInit	rs172032	ELL	C	T	0.00901	-0.0142217	0.002	0.0218158	0.425447	0.425338	1.56E-08	0.514465	0.425338	20.3	sarcoidosis
SmkInit	rs1733756	PCDH15	G	A	0.0101	0.0271085	0.002	0.0216101	0.49503	0.453305	1.23E-10	0.209682	0.453305	25.5	sarcoidosis
SmkInit	rs17432775	WDPCP	T	C	-0.0105	0.0348591	0.002	0.0291213	0.224652	0.163789	2.55E-08	0.231294	0.163789	27.56	sarcoidosis

SmkInit	rs17594561	NRXN3	G	A	0.00966	0.0301846	0.002	0.0236651	0.416501	0.295768	1.27E-09	0.202135	0.295768	23.33	sarcoidosis
SmkInit	rs1899689	CADPS2	T	C	0.00909	0.0307852	0.002	0.0220422	0.379722	0.401058	1.72E-08	0.162519	0.401058	20.66	sarcoidosis
SmkInit	rs192477591	SLC24A3	T	C	0.0116	0.00777783	0.002	0.0217599	0.500994	0.445531	1.73E-08	0.720764	0.445531	33.64	sarcoidosis
SmkInit	rs1945737	TCF4	C	T	-0.00968	-0.0174506	0.002	0.0216922	0.514911	0.544821	8.19E-10	0.421129	0.455179	23.43	sarcoidosis
SmkInit	rs1971318	HNF1A	T	C	0.0156	0.0156964	0.002	0.0337006	0.129225	0.117029	7.80E-13	0.641387	0.117029	60.84	sarcoidosis
SmkInit	rs1994247	KCNJ3	T	G	0.0122	0.002678	0.002	0.0215822	0.514911	0.527067	1.25E-14	0.901249	0.472933	37.21	sarcoidosis
SmkInit	rs2022815	FOXG1	A	G	0.0104	0.0155952	0.002	0.0261616	0.335984	0.216065	1.12E-09	0.551102	0.216065	27.04	sarcoidosis
SmkInit	rs2072155	MAGI2	C	T	0.0128	-0.0165415	0.002	0.0260363	0.699801	0.782459	1.16E-13	0.525216	0.217541	40.96	sarcoidosis
SmkInit	rs2135160	ERBB4	C	T	0.0157	0.0675393	0.003	0.0420938	0.0914513	0.0705663	8.23E-09	0.108605	0.0705663	27.39	sarcoidosis
SmkInit	rs2163413	NYAP2	G	A	-0.0138	-0.0133424	0.002	0.0304234	0.175944	0.146052	3.06E-11	0.660983	0.146052	47.61	sarcoidosis
SmkInit	rs2173019	TENM2	A	T	0.0131	0.0173817	0.002	0.0314966	0.170974	0.135339	3.47E-10	0.581045	0.135339	42.9	sarcoidosis
SmkInit	rs2289791	SMAD3	T	G	-0.0128	-0.00251379	0.002	0.0265603	0.240557	0.210801	2.87E-12	0.924597	0.210801	40.96	sarcoidosis
SmkInit	rs2292239	ERBB3	G	T	0.0108	0.022767	0.002	0.0233363	0.666998	0.693071	1.03E-10	0.32926	0.306929	29.16	sarcoidosis
SmkInit	rs2313500	KIF4B	T	C	0.0128	0.00874349	0.002	0.0234603	0.244533	0.307159	1.99E-12	0.709377	0.307159	40.96	sarcoidosis
SmkInit	rs2402821	GRM8	A	G	0.0103	0.00519961	0.002	0.0227365	0.62326	0.659973	3.51E-09	0.819109	0.340027	26.52	sarcoidosis
SmkInit	rs2678903	VRK2	G	A	0.0104	0.00206993	0.002	0.0222454	0.591451	0.622347	1.25E-10	0.925864	0.377653	27.04	sarcoidosis
SmkInit	rs2708630	RERE	T	C	-0.0102	0.0331161	0.002	0.0223772	0.691849	0.634232	9.99E-10	0.1389	0.365768	26.01	sarcoidosis
SmkInit	rs2711607	WDR72	T	G	0.0114	0.0111397	0.002	0.0265597	0.157058	0.208186	4.09E-08	0.674909	0.208186	32.49	sarcoidosis
SmkInit	rs2876586	UTRN	A	G	0.00905	-0.0208675	0.002	0.0221199	0.407555	0.381641	1.88E-08	0.345485	0.381641	20.48	sarcoidosis
SmkInit	rs288181	FBXL17	T	C	-0.00923	-0.00294218	0.002	0.0222952	0.317097	0.374604	4.11E-08	0.895012	0.374604	21.3	sarcoidosis
SmkInit	rs2939756	LRRC4C	A	G	-0.0103	-0.0135948	0.002	0.0216132	0.489066	0.511115	6.87E-11	0.529345	0.488885	26.52	sarcoidosis
SmkInit	rs3110590	DSC3	A	C	0.0111	0.00407294	0.002	0.0254096	0.248509	0.232535	1.22E-09	0.872652	0.232535	30.8	sarcoidosis
SmkInit	rs3213876	SMIM21	C	T	0.0115	0.00570672	0.002	0.0234861	0.315109	0.300052	5.08E-12	0.808019	0.300052	33.06	sarcoidosis
SmkInit	rs326341	CD47	A	G	-0.0101	0.00106095	0.002	0.02172	0.462227	0.446536	6.16E-10	0.961042	0.446536	25.5	sarcoidosis
SmkInit	rs332827	NFIA	A	G	-0.00893	0.0070655	0.002	0.0215526	0.452286	0.491442	2.81E-08	0.743043	0.491442	19.94	sarcoidosis
SmkInit	rs34367058	THSD7B	T	C	0.0138	-0.0242029	0.002	0.0270638	0.170974	0.198041	1.36E-13	0.371167	0.198041	47.61	sarcoidosis
SmkInit	rs34488670	SEMA6D	C	T	0.0176	-0.00585603	0.002	0.0258077	0.206759	0.22304	8.73E-20	0.820494	0.22304	77.44	sarcoidosis
SmkInit	rs35891966	NAV2	A	G	-0.019	-0.017047	0.003	0.0400879	0.0715706	0.0839921	6.52E-10	0.670661	0.0839921	40.11	sarcoidosis
SmkInit	rs3781295	GBF1	A	G	-0.0116	-0.0425578	0.002	0.0221127	0.387674	0.38604	1.13E-12	0.0542813	0.38604	33.64	sarcoidosis
SmkInit	rs3801289	DLX6	C	A	-0.0115	0.00873592	0.002	0.0217656	0.363817	0.433738	3.64E-12	0.688153	0.433738	33.06	sarcoidosis
SmkInit	rs3814994	ZNF804B	T	G	0.00962	-0.00918238	0.002	0.0227478	0.373757	0.339643	7.44E-09	0.686463	0.339643	23.14	sarcoidosis
SmkInit	rs3867541	NPIPA8	A	G	-0.0101	0.0202487	0.002	0.0222052	0.340954	0.418172	9.15E-09	0.361825	0.418172	25.5	sarcoidosis
SmkInit	rs3895907	FPGT-TNNI3K,TNNI3K	G	A	-0.0129	-0.0221125	0.002	0.021573	0.548708	0.491698	1.56E-14	0.30536	0.491698	41.6	sarcoidosis
SmkInit	rs3905125	ACTN2	T	C	0.0104	-0.00177164	0.002	0.0216355	0.542744	0.543657	4.28E-11	0.934738	0.456343	27.04	sarcoidosis
SmkInit	rs3934797	C4orf32	A	G	-0.0151	-0.0317519	0.002	0.0297929	0.171968	0.156001	1.90E-13	0.286535	0.156001	57	sarcoidosis
SmkInit	rs39784	RANBP17	A	C	0.0115	-0.018396	0.002	0.0230605	0.722664	0.675969	6.41E-11	0.425028	0.324031	33.06	sarcoidosis
SmkInit	rs404263	TMEM132B	T	C	-0.0112	-0.032699	0.002	0.0226338	0.65507	0.655859	1.55E-10	0.148543	0.344141	31.36	sarcoidosis
SmkInit	rs4044321	TENM2	G	A	-0.0164	0.0105148	0.002	0.0227799	0.668986	0.661373	3.04E-23	0.644381	0.338627	67.24	sarcoidosis
SmkInit	rs4374330	UBE2E3	T	C	0.0127	0.00449699	0.002	0.0254589	0.764414	0.765682	3.73E-12	0.859793	0.234318	40.32	sarcoidosis
SmkInit	rs4479577	EDEM1	T	C	0.00898	0.00485302	0.002	0.0215852	0.498012	0.501712	2.11E-08	0.822111	0.498288	20.16	sarcoidosis
SmkInit	rs4543592	PUM3	C	T	0.012	0.0150906	0.002	0.0220682	0.49006	0.393052	2.51E-14	0.494092	0.393052	36	sarcoidosis
SmkInit	rs4571506	TMEM161B	T	C	-0.0151	-0.0252582	0.002	0.0216538	0.481113	0.456521	1.64E-21	0.243429	0.456521	57	sarcoidosis
SmkInit	rs4579569	BHLHE22	A	G	0.0122	0.00647161	0.002	0.0216373	0.499006	0.543182	1.23E-14	0.764867	0.456818	37.21	sarcoidosis
SmkInit	rs465646	REV3L	A	G	-0.0236	0.0578459	0.002	0.0272162	0.849901	0.801455	1.70E-28	0.0335521	0.198545	139.24	sarcoidosis
SmkInit	rs4659805	RYR2	T	G	0.00936	-0.00624695	0.002	0.0233745	0.605368	0.693624	8.36E-09	0.789273	0.306376	21.9	sarcoidosis
SmkInit	rs4751614	AC023283.1,SHTN1	T	A	0.0122	0.025009	0.002	0.026965	0.23161	0.197265	6.35E-11	0.353687	0.197265	37.21	sarcoidosis
SmkInit	rs4785187	ZNF423	A	G	0.0119	0.00906261	0.002	0.0236958	0.244533	0.293311	2.03E-10	0.702123	0.293311	35.4	sarcoidosis
SmkInit	rs4819027	ADARB1	G	C	-0.00989	0.0232891	0.002	0.0250837	0.718688	0.753925	1.34E-08	0.353171	0.246075	24.45	sarcoidosis
SmkInit	rs4837631	BRINP1	T	C	-0.00949	0.0139442	0.002	0.0216488	0.457256	0.454336	3.54E-09	0.519505	0.454336	22.52	sarcoidosis
SmkInit	rs4857114	NSUN3	T	C	0.00881	-0.000540633	0.002	0.0220614	0.406561	0.395573	3.03E-08	0.980449	0.395573	19.4	sarcoidosis
SmkInit	rs4888444	TERF2IP	G	A	-0.0245	-0.114823	0.004	0.0820846	0.0397614	0.0171555	6.84E-10	0.161863	0.0171555	37.52	sarcoidosis
SmkInit	rs4944844	FAM168A	C	T	-0.0133	-0.0319964	0.002	0.0299936	0.84493	0.847429	2.33E-10	0.286073	0.152571	44.22	sarcoidosis
SmkInit	rs540356	NTM	A	C	0.0116	-0.0187589	0.002	0.0219159	0.370775	0.41096	4.07E-13	0.392024	0.41096	33.64	sarcoidosis
SmkInit	rs540860	SORL1	G	A	0.0117	-0.0244696	0.002	0.0217608	0.562624	0.548676	3.18E-13	0.260809	0.451324	34.22	sarcoidosis
SmkInit	rs551739	SLC2A13	T	A	0.0102	-0.0125777	0.002	0.024338	0.694831	0.730001	4.42E-09	0.605302	0.269999	26.01	sarcoidosis
SmkInit	rs557544	FOXO3	C	T	-0.00945	-0.000415119	0.002	0.0236109	0.356859	0.293984	2.82E-08	0.985973	0.293984	22.33	sarcoidosis
SmkInit	rs55921136	SYT14	C	T	-0.0136	-0.0105257	0.002	0.024165	0.240557	0.27356	1.90E-12	0.663144	0.27356	46.24	sarcoidosis
SmkInit	rs55942317	ZNF423	A	G	0.0173	0.0497646	0.003	0.0321163	0.0795229	0.129905	4.08E-11	0.121259	0.129905	33.25	sarcoidosis
SmkInit	rs56169608	IGSF21	A	G	0.00906	-0.00687865	0.002	0.0217454	0.441352	0.449761	0.00000001	0.751754	0.449761	20.52	sarcoidosis
SmkInit	rs56225373	ERCC4	G	T	0.016	0.000283307	0.003	0.0312164	0.158052	0.139742	3.13E-08	0.992759	0.139742	28.44	sarcoidosis
SmkInit	rs56348592	BNC2	G	A	0.0126	-0.0200254	0.002	0.0322238	0.142147	0.126489	1.81E-08	0.534305	0.126489	39.69	sarcoidosis
SmkInit	rs56820925	CBLN4	T	C	-0.0108	0.0243493	0.002	0.0224104	0.354871	0.362422	2.25E-10	0.277251	0.362422	29.16	sarcoidosis
SmkInit	rs5751239	TCF20	T	C	-0.0102	-0.0211931	0.002	0.0215548	0.527833	0.4625	1.02E-10	0.325498	0.4625	26.01	sarcoidosis
SmkInit	rs58400863	PCDH7	A	G	-0.0114	0.0107817	0.002	0.0225424	0.385686	0.354292	5.03E-12	0.632448	0.354292	32.49	sarcoidosis
SmkInit	rs58547189	PGPEP1	A	G	-0.0106	-0.0322795	0.002	0.0262706	0.245527	0.21398	1.73E-08	0.219172	0.21398	28.09	sarcoidosis
SmkInit	rs60453921	GRID1	A	T	0.0127	-0.00288353	0.002	0.0265901	0.150099	0.208668	1.06E-09	0.913644	0.208668	40.32	sarcoidosis
SmkInit	rs6088618	NCOA6	A	G	-0.0108	0.00655957	0.002	0.0215691	0.4334	0.475148	5.89E-11	0.761037	0.475148	29.16	sarcoidosis
SmkInit	rs6141314	AL034550.1,NOL4L	A	G	0.0131	-0.0107508	0.002	0.0227328	0.215706	0.34593	3.59E-13	0.636269	0.34593	42.9	sarcoidosis
SmkInit	rs61533748	KLHL29	C	T	0.0104	0.00146357	0.002	0.0217694	0.377734	0.440255	1.50E-10	0.946398	0.440255	27.04	sarcoidosis
SmkInit	rs61959481	PRR20A	A	G	-0.0123	-0.0176173	0.002	0.0281849	0.203777	0.178366	2.06E-10	0.531931	0.178366	37.82	sarcoidosis
SmkInit	rs62107261	FAM150B	C	T	-0.0259	-0.0863519	0.004	0.0774634	0.0447316	0.0199946	2.13E-11	0.26496	0.0199946	41.93	sarcoidosis
SmkInit	rs62135525	LRPPRC	T	C	-0.0221	0.0373755	0.004	0.0496398	0.0387674	0.0498909	4.61E-09	0.45149	0.0498909	30.53	sarcoidosis
SmkInit	rs62181771	SPATS2L	A	G	0.00989	-0.016811	0.002	0.021659	0.559642	0.478737	4.47E-10	0.437651	0.478737	24.45	sarcoidosis
SmkInit	rs62250713	CADM2	G	A	-0.0189	0.0380392	0.002	0.0239675	0.633201	0.71921	1.63E-30	0.112486	0.28079	89.3	sarcoidosis
SmkInit	rs62254171	CADM2	A	G	0.031	-0.0733449	0.005	0.0845051	0.0149105	0.0160327	3.73E-09	0.38543	0.0160327	38.44	sarcoidosis
SmkInit	rs62258903	CNTN3	G	C	-0.0158	-0.0222786	0.002	0.0328284	0.127237	0.124237	7.20E-12	0.497366	0.124237	62.41	sarcoidosis
SmkInit	rs62266876	COPG1	G	C	0.0154	0.0512728	0.003	0.0413262	0.0725646	0.071717	3.06E-08	0.214722	0.071717	26.35	sarcoidosis
SmkInit	rs62419578	SNAP91	A	T	0.0131	0.0306601	0.002	0.0298744	0.122266	0.153338	1.08E-08	0.30475	0.153338	42.9	sarcoidosis
SmkInit	rs6265	BDNF	T	C	-0.0199	0.00531727	0.002	0.029883	0.196819	0.154007	1.86E-22	0.858773	0.154007	99	sarcoidosis
SmkInit	rs6438208	ZBTB20	A	G	-0.0104	0.0481857	0.002	0.0243266	0.248509	0.271647	7.33E-09	0.0476157	0.271647	27.04	sarcoidosis
SmkInit	rs6464024	ELFN1	T	C	-0.0121	-0.0230671	0.002	0.0216575	0.437376	0.444562	3.38E-14	0.286837	0.444562	36.6	sarcoidosis
SmkInit	rs6472232	PDE7A	G	T	-0.00942	0.00383414	0.002	0.023967	0.375746	0.280967	1.43E-08	0.8729	0.280967	22.18	sarcoidosis
SmkInit	rs6497840	ZKSCAN2	A	G	0.0128	0.0144339	0.002	0.0246094	0.713718	0.743367	1.13E-12	0.557527	0.256633	40.96	sarcoidosis
SmkInit	rs6598539	IGF1R	C	T	0.011	0.0181603	0.002	0.0215604	0.512922	0.520986	5.76E-12	0.399621	0.479014	30.25	sarcoidosis
SmkInit	rs66680800	CADM2	T	G	-0.0125	-0.0126288	0.002	0.02208	0.430418	0.394609	1.13E-14	0.567351	0.394609	39.06	sarcoidosis
SmkInit	rs6728726	TMEM18	C	T	0.0187	-0.0175606	0.002	0.028884	0.83002	0.8352	4.27E-19	0.543207	0.1648	87.42	sarcoidosis
SmkInit	rs6852117	GALNTL6	G	C	-0.0118	-0.0120028	0.002	0.0226634	0.40159	0.341905	1.97E-13	0.59638	0.341905	34.81	sarcoidosis
SmkInit	rs6861333	ISL1	G	C	-0.0106	-0.014449	0.002	0.022178	0.357853	0.379421	3.12E-10	0.514725	0.379421	28.09	sarcoidosis
SmkInit	rs6868892	CDH12	T	C	0.0101	-0.00512707	0.002	0.0217134	0.50497	0.446777	1.92E-08	0.813336	0.446777	25.5	sarcoidosis
SmkInit	rs6959670	IMMP2L	T	C	0.00987	0.000115562	0.002	0.0226909	0.355865	0.347079	7.29E-09	0.995936	0.347079	24.35	sarcoidosis
SmkInit	rs7092291	CELF2	T	C	-0.00986	0.00846012	0.002	0.0226706	0.412525	0.342328	7.74E-10	0.709018	0.342328	24.3	sarcoidosis
SmkInit	rs71367545	KCNG2	A	G	0.0124	0.0499018	0.002	0.0262414	0.161034	0.216972	1.70E-10	0.0572176	0.216972	38.44	sarcoidosis
SmkInit	rs71491832	NRGN	G	C	-0.0183	0.029414	0.003	0.0362244	0.0745527	0.10077	3.72E-10	0.416795	0.10077	37.21	sarcoidosis
SmkInit	rs71627577	ZNF131	G	A	-0.017	0.0177152	0.002	0.0288214	0.119284	0.172401	5.15E-12	0.538783	0.172401	72.25	sarcoidosis
SmkInit	rs7195043	DEF8	T	C	-0.00883	0.00996052	0.002	0.0221148	0.444334	0.548374	2.13E-08	0.652423	0.451626	19.49	sarcoidosis
SmkInit	rs7205551	NFAT5	A	G	0.0102	-0.0176439	0.002	0.0217468	0.434394	0.425179	1.15E-09	0.417175	0.425179	26.01	sarcoidosis
SmkInit	rs7224742	C17orf75	T	C	-0.011	-0.0269622	0.002	0.0226457	0.633201	0.654903	1.02E-11	0.233806	0.345097	30.25	sarcoidosis
SmkInit	rs72664906	COL16A1	C	T	0.0143	-0.0349112	0.002	0.0287161	0.119284	0.17114	1.85E-09	0.224086	0.17114	51.12	sarcoidosis
SmkInit	rs72733235	ALDH1B1	C	T	0.0127	-0.0561788	0.002	0.025971	0.177932	0.220817	1.86E-09	0.0305309	0.220817	40.32	sarcoidosis
SmkInit	rs72789627	EFNA5	T	C	-0.0171	-0.0493845	0.002	0.0385551	0.134195	0.0844639	1.54E-13	0.200235	0.0844639	73.1	sarcoidosis
SmkInit	rs72904370	PHF21A	C	G	-0.0108	-0.0394848	0.002	0.0254296	0.217694	0.235672	1.59E-08	0.120493	0.235672	29.16	sarcoidosis
SmkInit	rs7333559	CLYBL	A	G	-0.014	0.0017557	0.002	0.0292417	0.791252	0.836121	3.24E-13	0.952123	0.163879	49	sarcoidosis
SmkInit	rs745570	CBX8	G	A	-0.0104	-0.0352688	0.002	0.0215374	0.49503	0.506093	8.70E-11	0.101513	0.493907	27.04	sarcoidosis
SmkInit	rs76132272	CDC42BPA	T	C	0.018	-0.0591782	0.003	0.0386283	0.055666	0.0854508	9.43E-09	0.125525	0.0854508	36	sarcoidosis
SmkInit	rs763053	WDR24	C	T	-0.0149	0.00208142	0.002	0.0226365	0.267396	0.349498	6.71E-16	0.926738	0.349498	55.5	sarcoidosis
SmkInit	rs76608582	CTB-50L17.10	A	C	-0.0267	-0.0336439	0.004	0.0469033	0.0457256	0.0570251	2.76E-13	0.473187	0.0570251	44.56	sarcoidosis
SmkInit	rs77307359	ARAP2	C	T	-0.0112	0.0389838	0.002	0.0269768	0.21173	0.198163	2.07E-08	0.148434	0.198163	31.36	sarcoidosis
SmkInit	rs7788527	WBSCR17	C	T	0.00952	-0.0163122	0.002	0.0233149	0.686879	0.694843	1.58E-08	0.484147	0.305157	22.66	sarcoidosis
SmkInit	rs7804551	ZKSCAN5	G	A	-0.0152	0.0405961	0.002	0.0260523	0.163022	0.217519	1.09E-12	0.119173	0.217519	57.76	sarcoidosis
SmkInit	rs78175438	BRWD1	C	T	0.0162	0.0262428	0.002	0.0326236	0.11829	0.123602	2.02E-11	0.42116	0.123602	65.61	sarcoidosis
SmkInit	rs7829715	TOX	C	T	-0.0117	0.0241287	0.002	0.021631	0.560636	0.547771	1.30E-13	0.26465	0.452229	34.22	sarcoidosis
SmkInit	rs79222572	C6orf118	G	T	0.0103	-0.0102328	0.002	0.0233292	0.255467	0.308366	1.53E-08	0.660932	0.308366	26.52	sarcoidosis
SmkInit	rs7929518	EED	G	A	0.0116	-0.0135396	0.002	0.0247241	0.772366	0.74191	9.06E-10	0.583949	0.25809	33.64	sarcoidosis
SmkInit	rs7947391	NPAS4	G	A	0.00929	-0.0252875	0.002	0.0226434	0.577535	0.654477	7.55E-09	0.264091	0.345523	21.58	sarcoidosis
SmkInit	rs7969559	CPSF6	G	A	-0.011	0.0136386	0.002	0.0225171	0.681909	0.644265	3.28E-10	0.544716	0.355735	30.25	sarcoidosis
SmkInit	rs7984311	PCCA	A	G	0.00921	0.0172176	0.002	0.0221287	0.387674	0.384184	1.77E-08	0.436532	0.384184	21.21	sarcoidosis
SmkInit	rs7986094	HMGB1	C	A	0.00988	-0.0242404	0.002	0.0226584	0.694831	0.653071	5.99E-09	0.2847	0.346929	24.4	sarcoidosis
SmkInit	rs8001839	HS6ST3	G	A	0.0094	-0.020762	0.002	0.0220365	0.323062	0.392639	3.74E-08	0.346108	0.392639	22.09	sarcoidosis
SmkInit	rs8067305	SMG6	A	G	0.011	0.03552	0.002	0.0216578	0.574553	0.53491	4.95E-12	0.100993	0.46509	30.25	sarcoidosis
SmkInit	rs8069451	FBXL20	C	T	0.0112	0.0462134	0.002	0.0283945	0.246521	0.173227	1.02E-09	0.103621	0.173227	31.36	sarcoidosis
SmkInit	rs888292	CDH2	T	A	-0.0107	0.0430651	0.002	0.0239524	0.295229	0.279696	2.03E-09	0.0721855	0.279696	28.62	sarcoidosis
SmkInit	rs911781	TACC2	G	A	-0.00878	0.0122927	0.002	0.0216136	0.568588	0.537269	4.99E-08	0.569527	0.462731	19.27	sarcoidosis
SmkInit	rs9323328	C14orf37	G	A	-0.0108	-0.0309722	0.002	0.0215843	0.526839	0.465557	7.25E-12	0.151303	0.465557	29.16	sarcoidosis
SmkInit	rs9375371	POU3F2	A	G	0.013	0.0159985	0.002	0.0229709	0.293241	0.328802	1.41E-13	0.486137	0.328802	42.25	sarcoidosis
SmkInit	rs9402093	LAMA2	T	G	0.0101	-0.000259591	0.002	0.0239546	0.691849	0.719646	3.38E-09	0.991354	0.280354	25.5	sarcoidosis
SmkInit	rs9423279	CPXM2	G	C	-0.0108	0.0223323	0.002	0.0220991	0.638171	0.598488	9.64E-11	0.312232	0.401512	29.16	sarcoidosis
SmkInit	rs951740	PTPRF	A	G	0.0172	-0.0285911	0.002	0.0220427	0.636183	0.606388	3.78E-26	0.194605	0.393612	73.96	sarcoidosis
SmkInit	rs9538536	DIAPH3	G	T	-0.00936	0.0241067	0.002	0.0224341	0.668986	0.637973	4.28E-08	0.282573	0.362027	21.9	sarcoidosis
SmkInit	rs9613472	MN1	G	A	0.00867	-0.0317105	0.002	0.0215051	0.541749	0.510172	4.02E-08	0.140331	0.489828	18.79	sarcoidosis
SmkInit	rs9627272	PRR34	C	G	-0.00991	0.00622053	0.002	0.0231002	0.389662	0.334491	1.28E-09	0.787711	0.334491	24.55	sarcoidosis
SmkInit	rs963354	PQLC2L	A	C	0.0107	-0.027469	0.002	0.0233501	0.704771	0.691419	2.35E-10	0.239435	0.308581	28.62	sarcoidosis
SmkInit	rs993700	CENPC	C	T	-0.0141	0.0110672	0.002	0.0255328	0.767396	0.767895	1.00E-13	0.664687	0.232105	49.7	sarcoidosis
SmkInit	rs9987376	RUNX1T1	G	T	-0.0137	-0.00521162	0.002	0.0224415	0.569583	0.642456	1.02E-17	0.816358	0.357544	46.92	sarcoidosis
LifeSmk	rs10052591	ISL1	T	C	0.012103282	-0.0144272	0.002020376	0.0219559	0.573358	0.589043	2.10E-09	0.511117	0.410957	35.89	sarcoidosis
LifeSmk	rs10226228	PDE1C	A	G	-0.016440558	0.0186606	0.002070606	0.0237654	0.62964	0.713208	2.00E-15	0.432337	0.286792	63.04	sarcoidosis
LifeSmk	rs10282292	IMMP2L	C	T	0.012913645	0.000961506	0.002084842	0.0222505	0.361838	0.374225	5.90E-10	0.965532	0.374225	38.37	sarcoidosis
LifeSmk	rs1050847	ZCCHC14	C	T	0.011484659	0.00563968	0.002024512	0.0216524	0.425845	0.493517	1.40E-08	0.794505	0.493517	32.18	sarcoidosis
LifeSmk	rs10879871	KCNC2	T	G	-0.013803677	-0.0147743	0.00210088	0.0225741	0.343436	0.350855	5.00E-11	0.512801	0.350855	43.17	sarcoidosis
LifeSmk	rs10918701	NOS1AP	G	A	0.011561646	0.00502916	0.002063243	0.0223358	0.372247	0.367167	2.10E-08	0.821853	0.367167	31.4	sarcoidosis
LifeSmk	rs11210229	LRRIQ3	A	G	0.016861446	0.0109533	0.002050016	0.0222414	0.383666	0.373464	2.00E-16	0.622385	0.373464	67.65	sarcoidosis
LifeSmk	rs112282219	HARBI1	G	A	-0.033380244	0.0733203	0.005049183	0.0675969	0.958949	0.9738863	3.80E-11	0.278068	0.0261137	43.71	sarcoidosis
LifeSmk	rs11255908	GATA3	T	G	-0.014511765	0.0479842	0.002288283	0.0292786	0.743485	0.840122	2.30E-10	0.101238	0.159878	40.22	sarcoidosis
LifeSmk	rs113382419	FAM163B	C	A	-0.040682308	-0.00514128	0.003189813	0.0599572	0.889325	0.9670792	3.00E-37	0.931666	0.0329208	162.66	sarcoidosis
LifeSmk	rs11768481	DLX6	C	A	0.012985964	-0.00785905	0.002124813	0.0217967	0.666168	0.568297	9.90E-10	0.718426	0.431703	37.35	sarcoidosis
LifeSmk	rs11783093	EPHX2	C	T	0.022636584	-0.0157026	0.002732744	0.0326305	0.838811	0.875375	1.20E-16	0.630356	0.124625	68.62	sarcoidosis
LifeSmk	rs11861214	FBXL16	G	T	0.013617671	-0.00854501	0.002427273	0.0227495	0.78364	0.659884	2.00E-08	0.707204	0.340116	31.48	sarcoidosis
LifeSmk	rs11948770	DNAH5	T	C	-0.014749658	0.0330996	0.002370631	0.0244448	0.768248	0.732923	4.90E-10	0.175719	0.267077	38.71	sarcoidosis
LifeSmk	rs12202536	EYS	A	G	-0.011864265	0.0361693	0.001997106	0.0216072	0.512754	0.538975	2.80E-09	0.0941413	0.461025	35.29	sarcoidosis
LifeSmk	rs1221148	BRINP1	C	G	0.013205933	-0.010296	0.002027509	0.0219523	0.587062	0.589438	7.30E-11	0.639058	0.410562	42.42	sarcoidosis
LifeSmk	rs12244388	AS3MT,BORCS7-ASMT	G	A	-0.019092395	-0.0153016	0.002109669	0.0229096	0.661124	0.672774	1.40E-19	0.504189	0.327226	81.9	sarcoidosis
LifeSmk	rs1246265	SLC28A3	T	C	-0.012773979	0.00440528	0.002174423	0.0239733	0.30456	0.28126	4.20E-09	0.854203	0.28126	34.51	sarcoidosis
LifeSmk	rs12481282	CD40	G	C	-0.012886801	0.0306086	0.002232463	0.024157	0.722343	0.725025	7.80E-09	0.20513	0.274975	33.32	sarcoidosis
LifeSmk	rs12623702	FZD7	A	G	-0.01408105	0.0318761	0.002057422	0.021646	0.613375	0.533618	7.70E-12	0.140857	0.466382	46.84	sarcoidosis
LifeSmk	rs12708665	TNRC6A	A	G	-0.013093629	-0.0053918	0.002217348	0.0241786	0.284632	0.273513	3.50E-09	0.823536	0.273513	34.87	sarcoidosis
LifeSmk	rs12831617	SLC6A15	C	T	-0.013234102	-0.034773	0.002353196	0.0290011	0.764305	0.835917	1.90E-08	0.230519	0.164083	31.63	sarcoidosis
LifeSmk	rs12967855	CELF4	A	G	0.011799121	-0.0191121	0.002131585	0.024407	0.331225	0.266005	3.10E-08	0.433594	0.266005	30.64	sarcoidosis
LifeSmk	rs13009008	AC013461.1	A	G	0.012438911	0.0164896	0.002122868	0.0223246	0.327713	0.376159	4.60E-09	0.460133	0.376159	34.33	sarcoidosis
LifeSmk	rs13016665	VRK2	C	A	-0.012229591	-0.00935067	0.002034267	0.0215993	0.576725	0.526738	1.80E-09	0.665076	0.473262	36.14	sarcoidosis
LifeSmk	rs13153393	TENM2	A	G	-0.019805354	-0.0137727	0.0031315	0.0371509	0.883937	0.9066779	2.50E-10	0.710845	0.0933221	40	sarcoidosis
LifeSmk	rs13296519	MAPKAP1	G	T	-0.013978602	0.00308732	0.002044468	0.0221781	0.606385	0.620101	8.10E-12	0.889287	0.379899	46.75	sarcoidosis

LifeSmk	rs136233	OSBP2	A	G	-0.014345831	0.0457647	0.002549217	0.0285311	0.809084	0.827565	1.80E-08	0.108707	0.172435	31.67	sarcoidosis
LifeSmk	rs147412694	BRWD1	G	A	-0.016674993	-0.0281618	0.002807815	0.0318936	0.850009	0.868983	2.90E-09	0.37724	0.131017	35.27	sarcoidosis
LifeSmk	rs17309874	BDNF	G	A	-0.016267649	-0.00109551	0.002279998	0.0239942	0.740357	0.722169	9.70E-13	0.963583	0.277831	50.91	sarcoidosis
LifeSmk	rs17553262	PCGF5	A	C	-0.018346008	-0.107819	0.003143301	0.0406136	0.884605	0.9298085	5.30E-09	0.00793652	0.0701915	34.07	sarcoidosis
LifeSmk	rs17576594	TTC29	G	A	0.015778175	-0.023373	0.002235691	0.0237329	0.7235	0.711225	1.70E-12	0.324707	0.288775	49.81	sarcoidosis
LifeSmk	rs1922018	SDK1	C	T	0.01445629	0.00883098	0.0020716	0.0228644	0.364422	0.328712	3.00E-12	0.699323	0.328712	48.7	sarcoidosis
LifeSmk	rs1931263	RWDD3	G	T	-0.010963455	-0.00147674	0.001996803	0.021768	0.510321	0.574993	4.00E-08	0.945913	0.425007	30.15	sarcoidosis
LifeSmk	rs1933270	AGBL4	T	G	0.013282689	-0.0382864	0.002072134	0.0220885	0.363726	0.399973	1.50E-10	0.0830386	0.399973	41.09	sarcoidosis
LifeSmk	rs202645	TEF	A	G	-0.014639285	-0.0297005	0.002485514	0.0253988	0.202939	0.234143	3.90E-09	0.242257	0.234143	34.69	sarcoidosis
LifeSmk	rs2062882	NECAB1	G	A	-0.011686155	-0.0328729	0.002046053	0.0216706	0.586782	0.556002	1.10E-08	0.129283	0.443998	32.62	sarcoidosis
LifeSmk	rs2080870	NDUFAF2	A	T	0.012439444	-0.025611	0.00228033	0.0250386	0.258107	0.24556	4.90E-08	0.306374	0.24556	29.76	sarcoidosis
LifeSmk	rs2254710	CCDC167	C	A	0.012962881	-0.045215	0.002349896	0.0229066	0.236356	0.331432	3.50E-08	0.0483949	0.331432	30.43	sarcoidosis
LifeSmk	rs245774	RANBP17	A	G	-0.012991195	0.017771	0.002247405	0.0230334	0.271691	0.324009	7.40E-09	0.440392	0.324009	33.41	sarcoidosis
LifeSmk	rs2675638	C10orf107	G	A	0.012245614	0.0499936	0.002017509	0.0221929	0.58057	0.617084	1.30E-09	0.024279	0.382916	36.84	sarcoidosis
LifeSmk	rs2838834	POFUT2	C	T	-0.013493897	0.0291244	0.002182795	0.0247745	0.699459	0.746943	6.30E-10	0.239763	0.253057	38.22	sarcoidosis
LifeSmk	rs28485305	C15orf59	C	T	0.011536819	-0.00377483	0.002073272	0.0222242	0.631482	0.622046	2.60E-08	0.865126	0.377954	30.96	sarcoidosis
LifeSmk	rs2867112	TMEM18	T	G	0.021301155	-0.0101092	0.002719661	0.0290133	0.834596	0.837154	4.80E-15	0.727513	0.162846	61.34	sarcoidosis
LifeSmk	rs2890772	ZEB2	G	T	-0.019737344	0.000588347	0.002026947	0.0248732	0.413181	0.248569	2.10E-22	0.981129	0.248569	94.82	sarcoidosis
LifeSmk	rs2894808	GSTA4	T	A	-0.022043365	0.0552145	0.003733668	0.0461137	0.922121	0.9426437	3.50E-09	0.231169	0.0573563	34.86	sarcoidosis
LifeSmk	rs317021	ARAP2	T	A	-0.016666924	0.0392001	0.00258047	0.0275459	0.814287	0.810423	1.10E-10	0.154712	0.189577	41.72	sarcoidosis
LifeSmk	rs326341	CD47	G	A	0.013595236	-0.00106095	0.002006414	0.02172	0.524943	0.553464	1.20E-11	0.961042	0.446536	45.91	sarcoidosis
LifeSmk	rs329120	JADE2	C	T	0.013914857	0.0225314	0.002024598	0.0217515	0.580672	0.575692	6.30E-12	0.300269	0.424308	47.24	sarcoidosis
LifeSmk	rs34866095	SOX6	A	G	-0.012351391	0.00266228	0.002169395	0.0252116	0.686187	0.757712	1.20E-08	0.915902	0.242288	32.42	sarcoidosis
LifeSmk	rs348809	CDH26	A	G	-0.011936166	-0.0196814	0.002097983	0.023153	0.347637	0.315032	1.30E-08	0.395291	0.315032	32.37	sarcoidosis
LifeSmk	rs35169606	TNKS	T	G	0.012645753	-0.0399489	0.002080448	0.0225	0.612204	0.639609	1.20E-09	0.0758141	0.360391	36.95	sarcoidosis
LifeSmk	rs35175834	SEMA6D	G	A	-0.023635418	0.00638282	0.00244733	0.0257796	0.78844	0.776949	4.60E-22	0.80445	0.223051	93.27	sarcoidosis
LifeSmk	rs35343344	PGPEP1	C	A	0.013229563	0.0308503	0.002299595	0.0253702	0.732713	0.761738	8.80E-09	0.223982	0.238262	33.1	sarcoidosis
LifeSmk	rs359243	BCL11A	T	C	-0.012564313	0.0260545	0.002053849	0.0224671	0.392886	0.360468	9.50E-10	0.246181	0.360468	37.42	sarcoidosis
LifeSmk	rs369230	CPNE7	G	T	-0.013096107	-0.0324037	0.002177204	0.0225554	0.307565	0.362697	1.80E-09	0.150825	0.362697	36.18	sarcoidosis
LifeSmk	rs3742365	ZFYVE21	T	C	-0.015551521	-0.00457251	0.002040174	0.0217089	0.595136	0.56241	2.50E-14	0.833177	0.43759	58.1	sarcoidosis
LifeSmk	rs3811038	TTL	T	C	-0.013752928	0.0424823	0.002244134	0.0258177	0.723852	0.769771	8.90E-10	0.0998711	0.230229	37.56	sarcoidosis
LifeSmk	rs3896224	SORCS3	A	G	0.013871514	-0.0173024	0.002043085	0.0215798	0.585286	0.50819	1.10E-11	0.422677	0.49181	46.1	sarcoidosis
LifeSmk	rs421983	CADM2	T	C	0.012546561	0.00648706	0.001996529	0.0217003	0.519028	0.434277	3.30E-10	0.764987	0.434277	39.49	sarcoidosis
LifeSmk	rs4391802	METTL15	A	G	0.014868821	0.00730848	0.002201541	0.0252651	0.707464	0.760298	1.40E-11	0.772373	0.239702	45.61	sarcoidosis
LifeSmk	rs4473348	UBE2E3	A	T	-0.015031067	-0.00454966	0.002300762	0.0254691	0.249978	0.234329	6.40E-11	0.858224	0.234329	42.68	sarcoidosis
LifeSmk	rs4543592	PUM3	T	C	-0.01248172	-0.0150906	0.002001558	0.0220682	0.519912	0.606948	4.50E-10	0.494092	0.393052	38.89	sarcoidosis
LifeSmk	rs4571506	TMEM161B	C	T	0.011350006	0.0252582	0.002005333	0.0216538	0.539523	0.543479	1.50E-08	0.243429	0.456521	32.03	sarcoidosis
LifeSmk	rs4671357	BCL11A	T	C	-0.013604645	-0.00860176	0.002002725	0.0220676	0.518819	0.393553	1.10E-11	0.696691	0.393553	46.15	sarcoidosis
LifeSmk	rs4731925	CHCHD3	C	T	-0.011948529	-0.0271819	0.002147262	0.0237063	0.315607	0.28837	2.60E-08	0.251542	0.28837	30.96	sarcoidosis
LifeSmk	rs4814873	SLC24A3	C	T	0.013994251	-0.0130491	0.00235785	0.0251875	0.76659	0.757711	2.90E-09	0.604402	0.242289	35.23	sarcoidosis
LifeSmk	rs4949465	COL16A1	T	C	-0.016722398	0.0351968	0.002965205	0.0286914	0.869552	0.829248	1.70E-08	0.219921	0.170752	31.8	sarcoidosis
LifeSmk	rs4957528	EFNA5	A	C	-0.014585539	-0.00898082	0.002481408	0.0306334	0.208497	0.144946	4.20E-09	0.769393	0.144946	34.55	sarcoidosis
LifeSmk	rs549845	PTPRF	G	A	0.01622644	-0.0200912	0.002173703	0.0244486	0.301161	0.262756	8.30E-14	0.411207	0.262756	55.72	sarcoidosis
LifeSmk	rs57611503	PRSS36	G	A	0.011157545	-0.0527949	0.002032048	0.0216469	0.484578	0.510188	4.00E-08	0.0147316	0.489812	30.15	sarcoidosis
LifeSmk	rs6011779	CHRNA4	C	T	0.027543147	-0.0448447	0.002541234	0.0250526	0.191239	0.244254	2.30E-27	0.0734514	0.244254	117.47	sarcoidosis
LifeSmk	rs60952428	ADAT1	T	C	0.019323804	0.0063338	0.00348602	0.0371048	0.909149	0.9076265	3.00E-08	0.86446	0.0923735	30.73	sarcoidosis
LifeSmk	rs6119897	AL034550.1,NOL4L	G	A	-0.018449032	0.00682989	0.002344407	0.023061	0.761716	0.677979	3.60E-15	0.767104	0.322021	61.93	sarcoidosis
LifeSmk	rs61796681	PPARGC1A	A	T	-0.019331585	0.00356372	0.003526639	0.0366789	0.912365	0.904678	4.20E-08	0.922599	0.095322	30.05	sarcoidosis
LifeSmk	rs62098013	DCC	G	A	-0.012343682	-0.000759418	0.00209905	0.0226052	0.63991	0.648356	4.10E-09	0.9732	0.351644	34.58	sarcoidosis
LifeSmk	rs62135536	PPM1B	C	T	0.035077916	-0.0472369	0.005708353	0.0538316	0.968256	0.9575156	8.00E-10	0.380218	0.0424844	37.76	sarcoidosis
LifeSmk	rs62155874	FHL2	A	G	-0.024361777	0.0636407	0.003004945	0.0348287	0.87336	0.892414	5.20E-16	0.0676628	0.107586	65.73	sarcoidosis
LifeSmk	rs62175972	RBMS1	T	C	0.031334018	-0.0368477	0.005557245	0.0831537	0.965939	0.9834699	1.70E-08	0.657673	0.0165301	31.79	sarcoidosis
LifeSmk	rs624833	ADD1	T	G	0.013379749	-0.0157988	0.002166556	0.0226244	0.694697	0.643442	6.60E-10	0.484985	0.356558	38.14	sarcoidosis
LifeSmk	rs6598539	IGF1R	T	C	-0.011744165	-0.0181603	0.002002005	0.0215604	0.488672	0.479014	4.50E-09	0.399621	0.479014	34.41	sarcoidosis
LifeSmk	rs6741228	KLHL29	T	C	0.011430423	0.012897	0.002022495	0.0216453	0.433309	0.526099	1.60E-08	0.551286	0.473901	31.94	sarcoidosis
LifeSmk	rs67596067	CA10	G	A	-0.012790405	0.0118613	0.002101456	0.0238942	0.648812	0.717199	1.20E-09	0.619605	0.282801	37.04	sarcoidosis
LifeSmk	rs6778080	USP4	T	C	0.016014339	-0.0294888	0.002256757	0.0262275	0.267372	0.214482	1.30E-12	0.260865	0.214482	50.36	sarcoidosis
LifeSmk	rs6779302	PLCL2	G	T	-0.012611546	-0.0663804	0.002073747	0.0222374	0.632921	0.640057	1.20E-09	0.00283511	0.359943	36.98	sarcoidosis
LifeSmk	rs6935954	HIST1H2BH	A	G	0.013806299	0.0245454	0.002020031	0.0226382	0.421108	0.344127	8.20E-12	0.278256	0.344127	46.71	sarcoidosis
LifeSmk	rs6957896	PLXNA4	C	T	-0.010928297	0.0305218	0.00199846	0.021952	0.503261	0.58594	4.50E-08	0.164412	0.41406	29.9	sarcoidosis
LifeSmk	rs6962772	ZNF789	A	G	0.015942149	-0.0355708	0.002761418	0.0258101	0.845551	0.776458	7.80E-09	0.16815	0.223542	33.33	sarcoidosis
LifeSmk	rs7039819	TLE4	G	A	0.012585321	0.0208931	0.002024483	0.0217006	0.427307	0.450006	5.10E-10	0.335652	0.450006	38.65	sarcoidosis
LifeSmk	rs7077678	ARL3	C	T	0.01232008	-0.00836707	0.002068776	0.0221201	0.623429	0.609204	2.60E-09	0.70524	0.390796	35.47	sarcoidosis
LifeSmk	rs71367545	KCNG2	G	A	-0.014869685	-0.0499018	0.00245501	0.0262414	0.790533	0.783028	1.40E-09	0.0572176	0.216972	36.69	sarcoidosis
LifeSmk	rs7155595	IRF2BPL	A	C	-0.012754325	-0.0228988	0.002139856	0.0235089	0.674282	0.69752	2.50E-09	0.33003	0.30248	35.53	sarcoidosis
LifeSmk	rs71627581	ZNF131	G	A	0.019101473	-0.0167333	0.003168128	0.0288683	0.888811	0.828376	1.60E-09	0.562156	0.171624	36.35	sarcoidosis
LifeSmk	rs72674867	KIAA1429	A	T	0.012950345	0.00401336	0.002354378	0.0223444	0.764568	0.627321	3.80E-08	0.857456	0.372679	30.26	sarcoidosis
LifeSmk	rs72678864	C4orf32	G	A	0.017842556	0.0270111	0.002649388	0.0312555	0.828591	0.861148	1.60E-11	0.387476	0.138852	45.35	sarcoidosis
LifeSmk	rs7297175	ERBB3	T	C	-0.011695074	-0.0509987	0.002016111	0.0217384	0.431355	0.429404	6.60E-09	0.0189754	0.429404	33.65	sarcoidosis
LifeSmk	rs732083	PGAP3	G	A	0.012028989	0.0496166	0.002122608	0.0230997	0.333362	0.318784	1.50E-08	0.031719	0.318784	32.12	sarcoidosis
LifeSmk	rs73220544	NEK11	A	C	-0.015588984	-0.0180151	0.002755871	0.0250166	0.842295	0.752232	1.50E-08	0.471447	0.247768	32	sarcoidosis
LifeSmk	rs7333559	CLYBL	G	A	0.015475297	-0.0017557	0.002459852	0.0292417	0.211711	0.163879	3.20E-10	0.952123	0.163879	39.58	sarcoidosis
LifeSmk	rs74086911	PRPF40B	G	A	0.021328389	-0.0253694	0.003806822	0.0470192	0.925459	0.9451092	2.10E-08	0.589504	0.0548908	31.39	sarcoidosis
LifeSmk	rs7519626	PLPPR5	C	T	0.012132618	-0.00368898	0.002130994	0.0221361	0.323676	0.38369	1.20E-08	0.867646	0.38369	32.41	sarcoidosis
LifeSmk	rs7528604	PDE4B	G	A	0.013908589	-0.0599428	0.002019296	0.0220653	0.565613	0.626939	5.70E-12	0.00659538	0.373061	47.44	sarcoidosis
LifeSmk	rs7553348	FPGT-TNNI3K,TNNI3K	G	A	0.013881644	0.02181	0.00201212	0.0215721	0.437656	0.508433	5.20E-12	0.312003	0.491567	47.6	sarcoidosis
LifeSmk	rs7569203	SIX3	A	C	-0.015503395	-0.000138155	0.002161643	0.022026	0.688725	0.599769	7.40E-13	0.994995	0.400231	51.44	sarcoidosis
LifeSmk	rs75742406	RPS13	G	A	0.013860736	0.0380321	0.002283629	0.0241122	0.738854	0.722311	1.30E-09	0.114727	0.277689	36.84	sarcoidosis
LifeSmk	rs76608582	CTB-50L17.10	C	A	0.031169179	0.0336439	0.004956749	0.0469033	0.953011	0.9429749	3.20E-10	0.473187	0.0570251	39.54	sarcoidosis
LifeSmk	rs7766610	REV3L	C	A	0.018132178	-0.0158352	0.002583294	0.0238844	0.182634	0.286833	2.20E-12	0.507335	0.286833	49.27	sarcoidosis
LifeSmk	rs7807019	CTTNBP2	A	G	-0.015015793	-0.000793648	0.002003748	0.0218739	0.540327	0.418817	6.70E-14	0.971057	0.418817	56.16	sarcoidosis
LifeSmk	rs8042134	SPATA8	T	G	-0.014324534	0.0285916	0.002018849	0.0216266	0.541083	0.509149	1.30E-12	0.186148	0.490851	50.34	sarcoidosis
LifeSmk	rs8042849	HYKK	C	T	0.027688966	-0.0553838	0.002106355	0.0225886	0.34229	0.355367	1.80E-39	0.0142125	0.355367	172.8	sarcoidosis
LifeSmk	rs860326	OTX2	C	T	0.012014363	0.0290588	0.002020578	0.0217075	0.427696	0.563436	2.70E-09	0.180684	0.436564	35.35	sarcoidosis
LifeSmk	rs8614	NUFIP2	C	A	-0.016514476	-0.0527575	0.002589	0.0298373	0.817489	0.84539	1.80E-10	0.0770318	0.15461	40.69	sarcoidosis
LifeSmk	rs889398	NFAT5	C	T	0.013314951	0.0069321	0.002036961	0.021903	0.588005	0.574307	6.30E-11	0.75163	0.425693	42.73	sarcoidosis
LifeSmk	rs9435340	PRMT6	T	A	0.012028268	-0.0128069	0.002109828	0.0236486	0.344234	0.296008	1.20E-08	0.588128	0.296008	32.5	sarcoidosis
LifeSmk	rs9842947	PQLC2L	C	T	-0.012639442	0.0284183	0.002133588	0.0233644	0.326165	0.308637	3.10E-09	0.223868	0.308637	35.09	sarcoidosis
LifeSmk	rs986391	TENM2	G	A	0.016049929	-0.0106535	0.002071874	0.0227062	0.366555	0.342877	9.40E-15	0.638935	0.342877	60.01	sarcoidosis
LifeSmk	rs9904288	GIP	T	C	0.012143324	-0.0139219	0.002194351	0.0242822	0.707772	0.733052	3.10E-08	0.566417	0.266948	30.62	sarcoidosis
LifeSmk	rs9919670	NCAM1	G	A	-0.021964835	0.000977223	0.002047725	0.0223471	0.612185	0.374492	7.60E-27	0.96512	0.374492	115.06	sarcoidosis

Abbreviations:
SNP, Single-nucleotide polymorphisms; SmkIni, initiation of smoking; AgeSmk, age of initiation of smoking; CigDay cigarettes per day; SmkCes, smoking cessation;

Table S8. The association of genetically predicted current tobacco smoking (CurSmk) and past tobacco smoking (PastSmk) with sarcoidosis

Exposure	Method	*P*	OR (95% CI)
CurSmk	Inverse variance weighted	5.89E-01	0.72 (0.22, 2.35)
F>=30.10	MR-Egger	8.10E-02	0.02 (0.00, 1.42)
nSNP=34	Weighted median	6.23E-01	0.68 (0.15, 3.17)
	MR-PRESSO †	6.22E-01	0.75 (0.24, 2.36)
PastSmk	Inverse variance weighted	3.32E-01	1.19 (0.83, 1.71)
F>=30.10	MR-Egger	6.97E-01	0.73 (0.15, 3.48)
nSNP=98	Weighted median	2.07E-01	1.29 (0.87, 1.94)
	MR-PRESSO *	1.00E-01	1.25 (0.96, 1.64)

† No outlier indicated by MR-PRESSO test

* removing rs1611723 and rs597808

Table S11. The heterogeneity and pleiotropy test for the association of genetically predicted current tobacco smoking (CurSmk) and past tobacco smoking (PastSmk) with sarcoidosis

Smoking	I^2	heterogeneity	pleiotropy		
		Q_pval	egger_intercept	MR Egger *P*	[R PRESSO *P*
CurSmk	21.43%	0.1385784	0.03190347	0.09577884	0.1676667
PastSmk	45.63%	1.90988E-06	0.009684118	0.5291486	<0.0001
PastSmk removing rs1611723 and rs597808	0.00%	0.7380888	-0.001554738	0.8912183	0.749

Table S9. The association of genetically predicted cotinine levels (COT) and cotinine plus 3'-hydroxycotinine levels (COT+3H) in current smokers with sarcoidosis

**Exposure**	**Method**	***P***	**OR (95% CI)**
Cotinine	Inverse variance weighted	5.95E-02	0.84 (0.69, 1.00)
F>=45.77	MR-Egger	8.39E-01	1.08 (0.60, 1.96)
nSNP=3	Weighted median	**5.88E-03**	**0.78 (0.65, 0.92)**
COT+3H	Inverse variance weighted	3.42E-01	0.89 (0.69, 1.14)
F>=31.41	MR-Egger	8.38E-01	1.24 (0.24, 6.31)
nSNP=3	Weighted median	1.85E-01	0.89 (0.75, 1.06)

Table S12. The association of genetically predicted cotinine levels (COT) and cotinine plus 3'-hydroxycotinine levels (COT+3H) in current smokers with sarcoidosis

**Smoking**	**I^2**	**heterogeneity**	**pleiotropy**	
		**Q_pval**	**egger_intercept**	**MR Egger *P***
Cotinine	39.80%	0.1900942	-0.05732652	0.5323443
COT+3H	62.52%	0.06939431	-0.08431432	0.7504405

COT+3H: Cotinine and 3'-hydroxycotinine levels

Table S10 The heterogeneity and pleiotropy test for the association of smoking behavior traits with sarcoidosis

**Smoking**	**I^2**	**heterogeneity Q_pval**	**pleiotropy**		
			egger intercept	MR Egger P	MR PRESSO P
LifeSmk	18.86%	**0.04461912**	0.02258742	**0.02930835**	**0.047**
LifeSmk removing rs6779302	13.69%	0.1165167	0.0204971	**0.04222705**	0.118
AgeSmk	61.14%	**0.0058515**	-0.07763855	0.2077188	**0.047**
AgeSmk removing rs12575642	0.00%	0.7373172	-0.06814098	0.1086251	0.7196
CigDay	22.88%	0.09181849	0.01196622	0.1460047	0.0649
SmkCes	0.00%	0.796742	0.01531861	0.3649216	0.7937
SmkIni	0.00%	0.9477344	0.003956635	0.6337518	0.9496

Abbreviations: SmkIni, initiation of smoking; AgeSmk, age of initiation of smoking; CigDay cigarettes per day; SmkCes, smoking cessation; LifeSmk, lifetime smoking index; MR-PRESSO, MR pleiotropy residual sum and outlier.
